# Myeloid cell reprogramming drives enhanced defense against *Streptococcus pneumoniae* lung infection following exposure to commensal *Prevotella*

**DOI:** 10.1073/pnas.2623525123

**Published:** 2026-10-07

**Authors:** Sara N. Stoner, Eric D. Larson, Sam Fulte, Steven C. Shaw, Erin R. Fish, Edward N. Janoff, Matthias Mack, Sarah E. Clark

**Affiliations:** ^a^ Department of Otolaryngology—Head and Neck Surgery, University of Colorado School of Medicine, Aurora, CO 80045; ^b^ Department of Basic and Translational Sciences, University of Pennsylvania School of Dental Medicine, Philadelphia, PA 19104; ^c^ Division of Infectious Diseases, University of Colorado School of Medicine, Aurora, CO 80045; ^d^Rocky Mountain Regional Veterans Affairs Medical Center, Aurora, CO 80045; ^e^ Department of Nephrology, University Hospital Regensburg, Regensburg 93053, Germany

**Keywords:** *Streptococcus pneumoniae*, lung infection, *Prevotella*, single cell RNA sequencing, lung microbiome

## Abstract

Clinical data link the prevalent respiratory tract anaerobe *Prevotella* with reduced pneumonia mortality, but the mechanisms directing *Prevotella* regulation of lung immune homeostasis are unclear. Here, single-cell RNA sequencing was employed to define the transcriptional immune signatures underlying improved clearance of *Streptococcus pneumoniae* following lung exposure to *Prevotella melaninogenica*. Overall, we observed a substantial shift in myeloid cell transcriptional programming from interferon-dominant to a more antibacterial profile in *S. pneumoniae*–infected mice after pre-exposure to *P. melaninogenica*, correlating with increased macrophage and neutrophil phagocytosis of *S. pneumoniae* and improved pathogen clearance. In neutrophils, tumor necrosis factor (TNF) signaling through TNFR2 was essential for increased antimicrobial function. Moreover, improved defense required CCR2-dependent monocyte-derived macrophages, with selective enrichment of more a mature Cxcl3+ population which was distinct from the hallmark *S. pneumoniae*–associated C1qa+ population enriched in the absence of effective clearance. Together, these findings inform the myeloid cell transcriptional changes associated with natural infection resistance mediated by pulmonary microbial exposures.

## Significance

Even though *Streptococcus pneumoniae* is the most common cause of bacterial lung infections, most people who are exposed to this bacterium can successfully defend against infection. We investigated a mechanism for this natural resilience against infection by exploring how the immune system is educated by lung exposures to a common airway bacterium, *Prevotella*. We found that *Prevotella* alters the protective functions of immune cells recruited to the lungs after *S. pneumoniae* challenge, resulting in improved immune-mediated bacterial clearance. This study reveals the cell type–specific transcriptional changes which occur in the lungs during *S. pneumoniae* infection, and how these are altered in a setting of improved infection defense following *Prevotella* exposure.

Growing evidence points to the respiratory tract microbiome as a key determinant of pneumonia outcomes (1–3). The upper respiratory tract acts as a microbial reservoir, with commensal bacteria from the oropharynx serving as the primary source of transient microbial exposures in the lower airway (4, 5). Greater microbial diversity in the upper and lower airway correlates with improved survival in pneumonia patients (6–8), whereas depletion of obligate anaerobes, including oral commensals (6), through antibiotic treatment or chronic oxygen therapy is linked to worse outcomes (9).

*Prevotella* species are core members of the oral microbiome and are consistently among the most abundant bacterial taxa detected in the lungs of healthy individuals (4, 10). Higher *Prevotella* abundance is inversely associated with respiratory pathogens in both the upper airway (8, 11) and the lungs (4, 12, 13). Specifically, the species *Prevotella melaninogenica* is enriched in healthy individuals compared to patients suffering from pneumonia, acute respiratory distress syndrome, and exacerbations of chronic obstructive pulmonary disease (11, 14, 15). In one report, *P. melaninogenica* was the most discriminatory species distinguishing healthy subjects from those with *Streptococcus pneumoniae* (pneumococcal) pneumonia (8). Beyond correlations with health, *Prevotella* are increasingly recognized as immunomodulatory. Bronchoalveolar lavage (BAL) samples enriched with *Prevotella* exhibit increased numbers of activated neutrophils and higher Th17 cell frequencies (16, 17), and lung transplant recipients with more diverse microbial profiles containing *Prevotella* have elevated expression of IL-10 (18). A single dose of *P. melaninogenica*, in combination with two other oral commensals, increased airway clearance of *S. pneumoniae* from the lungs of mice at 24 h and up to 14 d postexposure to the commensal mixture (19). These findings suggest that *Prevotella* may shape lung immune tone and infection susceptibility.

To mechanistically address the link between *Prevotella* and improved pneumonia outcomes, we previously determined that pre-exposure to live or heat-killed *P. melaninogenica* promoted rapid clearance of *S. pneumoniae* from the lungs (20), with a similar effect observed against *Staphylococcus aureus*, demonstrating a protective function against multiple respiratory pathogens (21). Here, scRNA-seq was used to define the transcriptional immune landscape during *S. pneumoniae* infection in susceptible vs. resistant hosts, with resistance modeled by improved pathogen clearance following *P. melaninogenica* priming.

## Results

### *P. melaninogenica* Exposure Alters the Myeloid Cell Response to Pneumococcal Pneumonia.

**Fig. 1.**
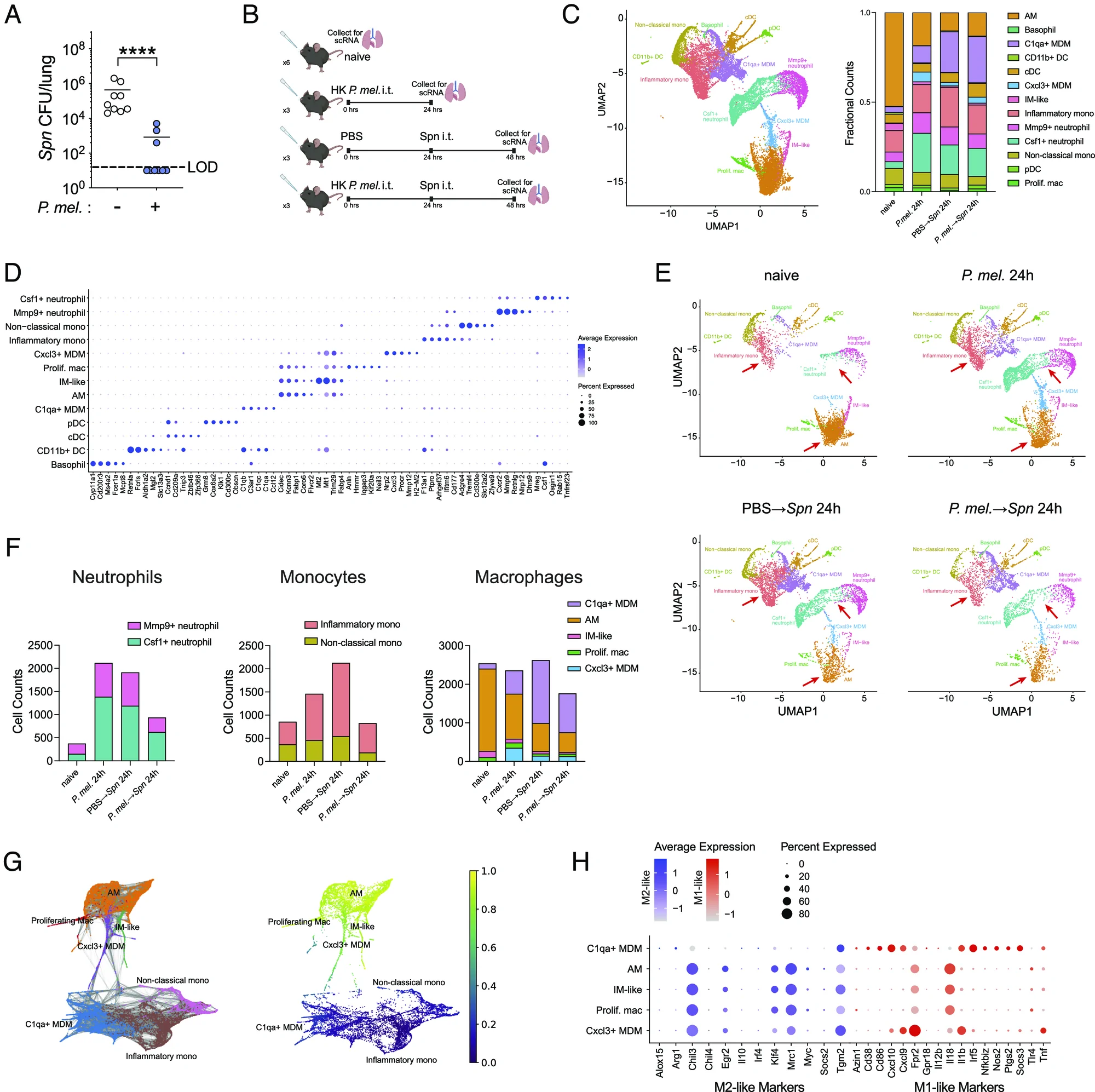
*P. melaninogenica* exposure alters the myeloid cell response to pneumococcal pneumonia. (*A*) *Spn* burdens in lung tissue of mice with exposure to PBS or *P. mel* HK 24 h prior to infection (mean ± SEM; n = 9 mice/group from three independent experiments). (*B*) ScRNA-seq experiment sample collection (n = 6 mice/naïve, n = 3 mice/all other groups). (*C*) UMAP projection of myeloid cell clusters and bar plot displaying relative proportions of myeloid populations. (*D*) Dot plot of top 5 differentially expressed genes (DEGs) in myeloid cell clusters. (*E*) UMAP projections of myeloid cell clusters split by condition; red arrows indicate notable changes. (*F*) Cell counts of neutrophil, monocytes, and macrophage populations detected in scRNA-seq samples. (*G*) ForceAtlas2 projection of monocyte and macrophage populations (*Left*) and PAGA-inferred pseudotime values (*Right*). (*H*) Expression of M1-like and M2-like marker genes across macrophage populations. Abbreviations: AM = alveolar macrophage, IM = interstitial macrophage, MDM = monocyte-derived macrophage, mono = monocyte, mac = macrophage, DC = dendritic cell, cDC = conventional DC, pDC = plasmacytoid DC. Data were analyzed by the Mann–Whitney test (*A*), ****P < 0.0001.

Mice primed with heat-killed (HK) *P. melaninogenica* 24 h prior to *S. pneumoniae* challenge had significantly reduced *S. pneumoniae* burdens at 24 h postinfection compared to unprimed mice (Fig. 1*A*). Single-cell RNA sequencing was performed on lung tissue of naïve mice, *P. melaninogenica*-exposed mice, and *S. pneumoniae*–infected mice with or without prior exposure to *P. melaninogenica* (Fig. 1*B*). Clustering analysis identified 30 distinct cell populations in lung tissue (*SI Appendix*, Fig. S1 *A*–*D*). Myeloid cell populations included monocytes, macrophages, neutrophils, dendritic cells, and basophils (Fig. 1 *C* and *D*). Broad changes among several myeloid cell populations were apparent in response to both *S. pneumoniae* infection and *P. melaninogenica* exposure, including large expansions in recruited monocytes and neutrophils with corresponding reductions in tissue-resident macrophages (Fig. 1*E*). Two neutrophil populations were detected, both of which were increased in *P. melaninogenica*-exposed and *S. pneumoniae*–infected mice compared to naïve mice. Recruited immune cells detected at baseline in naïve mice are likely in the marginated vascular compartment of the lungs, as reported elsewhere (22). Neutrophil recruitment increased in both *P. melaninogenica*-exposed and *S. pneumoniae*–infected mice, but was lower in mice pre-exposed to *P. melaninogenica* with enhanced *S. pneumoniae* clearance (Fig. 1*F*). Inflammatory monocytes were recruited to the lung tissue in *P. melaninogenica*-exposed mice and in even greater numbers in *S. pneumoniae*–infected mice (Fig. 1*F*). As with neutrophils, numbers of recruited inflammatory monocytes were lower with dual-bacterial exposure than with either alone. Tissue-resident alveolar macrophages (AMs), prominent at baseline, were depleted in both *P. melaninogenica*-exposed and *S. pneumoniae*–infected mice, alone and in combination, as anticipated following an acute inflammatory stimulus (23, 24), but more subtly with *P. melaninogenica* alone (Fig. 1*F*). A minority population of macrophages with high expression of cell division genes was present in all conditions (*SI Appendix*, Fig. S2), consistent with prior reports of a proliferative AM subpopulation (24).

Of note, two distinct populations of monocyte-derived macrophages (MDM) were identified which differed proportionally between groups; a prominent C1qa+ MDM population selectively enriched in *S. pneumoniae*–infected mice and a smaller Cxcl3+ MDM population predominantly enriched in *P. melaninogenica*-exposed mice (Fig. 1*F*). Partition-based graph abstraction (PAGA) clustering and pseudotime analysis revealed that C1qa+ MDMs clustered closely with immature monocytes, whereas Cxcl3+ MDMs clustered closer to the mature, tissue resident-like macrophages (Fig. 1*G*). Additionally, the prominent C1qa+ MDMs expressed high levels of pro-inflammatory “M1-like” signature genes, while the Cxcl3+ MDMs and tissue resident-like macrophage populations showed greater expression of pro-resolution “M2-like” signature genes (Fig. 1*H*). This pattern aligns with reports indicating that recruited macrophage populations exhibit an M1-like phenotype, and tissue-resident macrophages are more M2-like during acute lung injury (24, 25).

Given our prior finding that toll-like receptor 2 (TLR2) signaling was required for *P. melaninogenica–*induced neutrophil activation and lung clearance of *S. pneumoniae* (20), we examined *Tlr2* expression patterns. *Tlr2* was primarily expressed in myeloid cells (*SI Appendix*, Fig. S3*A*), focusing our analysis on myeloid cell types to identify gene expression pathways associated with *P. melaninogenica*-mediated protection. Unbiased pathway enrichment analysis of *S. pneumoniae*–infected mice with vs. without *P. melaninogenica* priming revealed distinctly enriched immune activation pathways, including TNF signaling and antigen presentation, in myeloid cells (*SI Appendix*, Fig. S3*B*). Differential signaling pathway enrichment was also apparent among other nonmyeloid cell types including epithelial cells and adaptive immune cells (*SI Appendix*, Fig. S3*C*).

### Immune Priming by *P. melaninogenica* Induces a Coordinated Recruitment of Myeloid Cells.

**Fig. 2.**
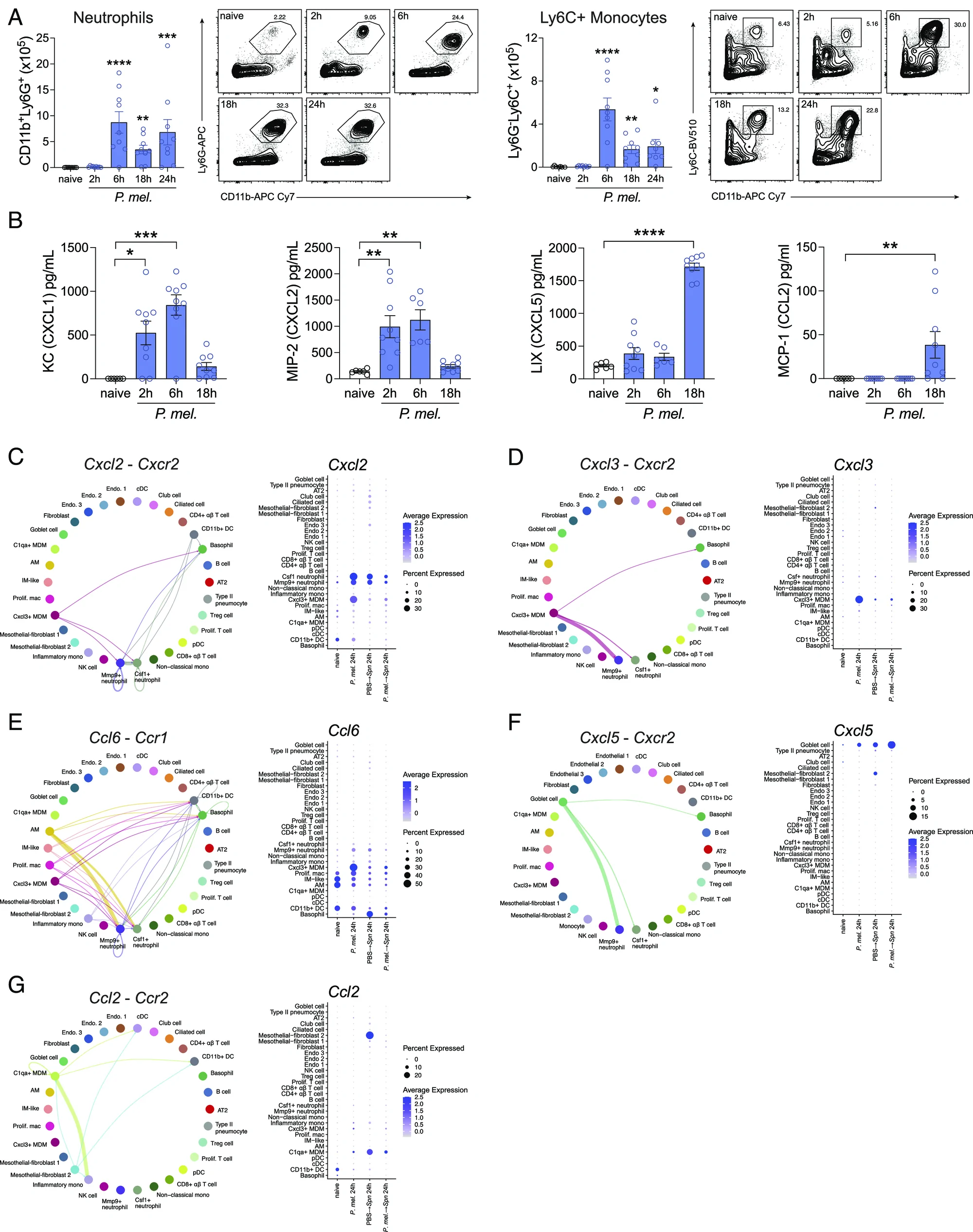
Immune priming by *P. melaninogenica* induces a coordinated recruitment of myeloid cells. (*A*) Quantification of CD11b+Ly6G+ neutrophils and Ly6G-Ly6C+ monocytes/macrophages in the lung tissue via flow cytometry after i.t. exposure to *P. mel* HK (mean ± SEM; n = 8-9 mice/group from three independent experiments). (*B*) Chemokine detection in lung BAL after i.t. exposure to *P. mel* HK (mean ± SEM; n = 6-9 mice/group from three independent experiments). CellChat circle plots displaying inferred cell–cell communication networks for (*C*) *Cxcl2*-*Cxcr2*, (*D*) *Cxcl3*-*Cxcr2*, (*E*) *Ccl6*-*Ccr1*, (*F*) *Cxcl5*-*Cxcr2*, and (*G*) *Ccr2*-*Ccl2* signaling alongside dot plots indicating expression of corresponding chemokine genes. Data were analyzed by Kruskal–Wallis with Dunn’s post hoc test (*A* and *B*) or ordinary one-way ANOVA with Dunnett’s post hoc test (*B*), *P < 0.05, **P < 0.01, ***P < 0.001, ****P < 0.0001.

Myeloid cell recruitment over time was examined in *P. melaninogenica*-primed mice via flow cytometry (*SI Appendix*, Fig. S4*A*). Neutrophils and Ly6C+ monocytes and MDMs were recruited to the lung tissue as early as 6 h postexposure to *P. melaninogenica*, with persistence at 24 h (Fig. 2*A*). This cellular recruitment coincided with elevated concentrations of neutrophil-recruiting chemokines CXCL1, CXCL2, and CXCL5 as well as the monocyte chemoattractant CCL2 in lung BAL fluid (Fig. 2*B*). CellChat analysis, which predicts cell–cell communication networks, in all treatment groups combined revealed multiple significantly enriched chemokine signaling pathways. For neutrophil-recruiting chemokines, *Cxcl2-Cxcr2* and *Cxcl3-Cxcr2* signaling pathways were both significantly enriched, with the Cxcl3+ MDM population predicted as a major source of CXCL2 sensed by Cxcr2-expressing neutrophils. CellChat analysis also identified the *Ccl6-Ccr1* signaling pathway as significantly enriched, with several macrophage populations predicted to signal to Ccr1-expressing neutrophils (Fig. 2 *C*–*E*). *Cxcl1* signaling networks were not significantly enriched. Stratification by condition showed that *Cxcl2*, *Cxcl3*, and *Ccl6* were highly expressed in macrophage populations from *P. melaninogenica*-exposed mice, particularly the Cxcl3+ MDMs which were selectively enriched in this group, consistent with the high neutrophil cell counts observed in *P. melaninogenica*-exposed mice.

Neutrophils were predicted to engage in autocrine signaling, as they expressed both chemokines and their corresponding receptors (Fig. 2*C*), suggesting that neutrophils contribute to their own recruitment through positive feedback. *Cxcl5*, which encodes a neutrophil-recruiting chemokine produced by epithelial cells, was expressed by goblet cells and predicted to signal to *Cxcr2*-expressing neutrophils (Fig. 2*F*). Last, *Ccl2*, which encodes a monocyte-recruiting chemokine, was expressed by fibroblasts and C1qa+ MDMs, and was predicted to signal to *Ccr2*-expressing inflammatory monocytes (Fig. 2*G*). Condition-specific analysis revealed that fibroblasts from *S. pneumoniae*–infected mice expressed high levels of *Ccl2* (Fig. 2*G*), correlating with the increased inflammatory monocyte counts observed in these mice compared to other groups. Both the C1qa+ MDMs and Cxcl3+ MDMs also expressed the T cell–recruiting chemokines *Cxcl16*, *Cxcl10*, and *Cxcl9*, predicted to signal to *Cxcr6* and *Cxcr3*-expressing regulatory T cells (*SI Appendix*, Fig. S4 *B*–*D*). These data highlight a coordinated signaling program with predicted contributions from macrophages and epithelial cells to the recruitment of innate immune cells to the lungs following *P. melaninogenica* exposure.

### *P. melaninogenica* Induces Neutrophil Transcriptional Profiles Associated with Enhanced Phagocytosis and Killing of *S. pneumoniae*.

**Fig. 3.**
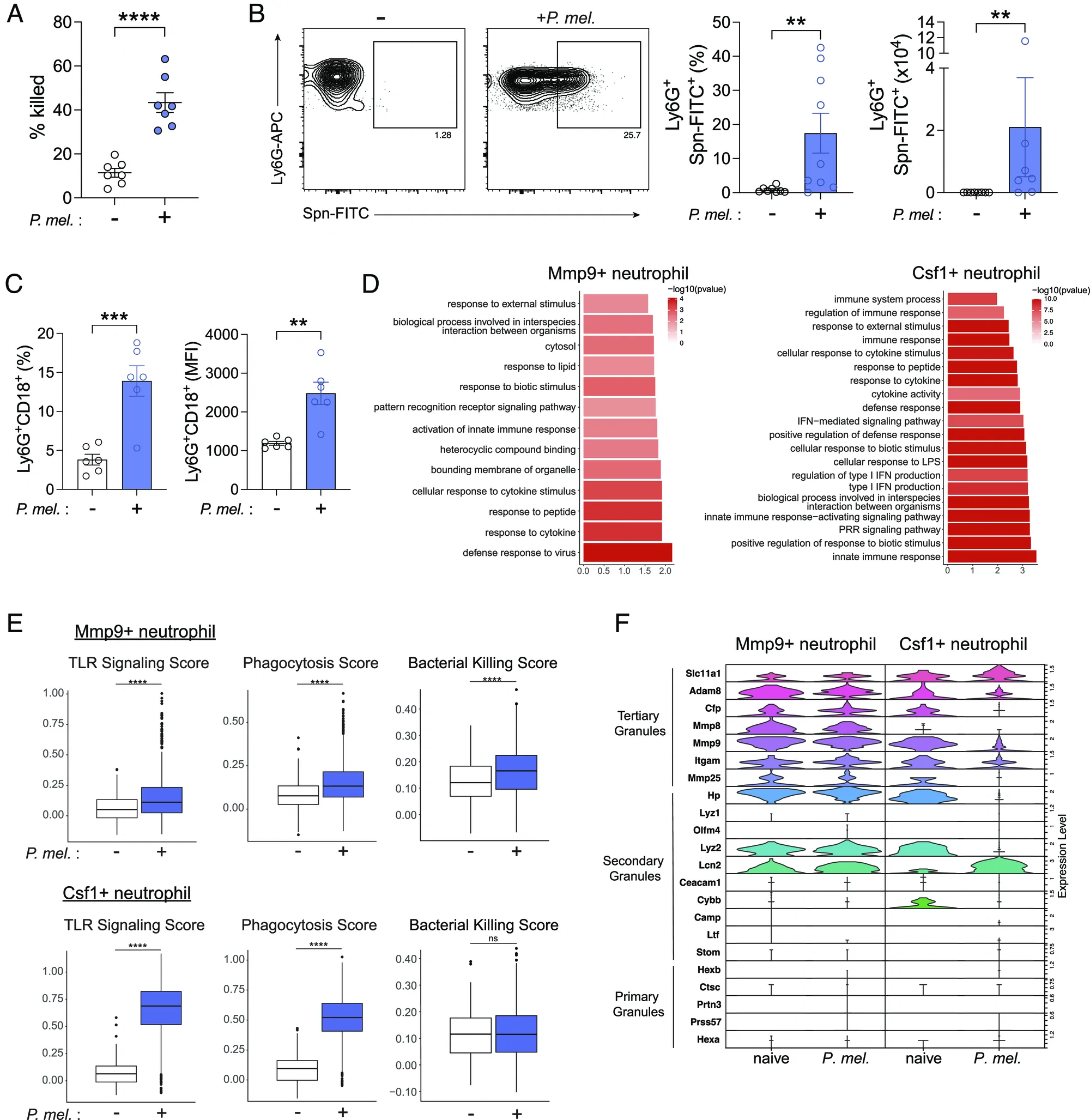
*P. melaninogenica* induces neutrophil transcriptional profiles associated with enhanced phagocytosis and killing of *S. pneumoniae*. (*A*) Percent of *Spn* killed by neutrophils isolated from mice exposed to either *E. coli* LPS or *P. mel* HK for 24 h (mean ± SEM; n = 7 mice/group from three independent experiments). Quantification of Ly6G+Spn-FITC+ (*B*) and Ly6G+CD18+ (*C*) neutrophils in lung tissue of mice exposed to FITC-labeled *Spn* HK for 3 h, after 24 h PBS or *P. mel* HK exposure (mean ± SEM; n = 6-9 mice/group from two to three independent experiments). (*D*) Top differentially enriched GO pathways in neutrophils of mice exposed to *P. mel* HK relative to naïve mice. (*E*) Module scoring analysis of indicated signaling pathways in neutrophils of naïve mice and mice exposed to *P. mel* HK. (*F*) Violin plot displaying expression of granule genes in neutrophils of naïve mice and mice exposed to *P. mel* HK. Data were analyzed by the unpaired *t* test (*A* and *C*), Mann–Whitney test (*B*), or Wilcoxon rank-sum test (*E*), **P < 0.01, ***P < 0.001, ****P < 0.0001.

We previously reported the unique capacity for *P. melaninogenica* to enhance *S. pneumoniae* clearance, as 24 h exposure to other commensal bacteria or *Escherichia coli* lipopolysaccharide (LPS) did not provide the same protective benefit (20). Here, we found that neutrophils purified from the lungs of *P. melaninogenica*-exposed mice exhibited an increased capacity to kill *S. pneumoniae* compared to neutrophils from mice treated with *E. coli* LPS (Fig. 3*A*). Neutrophil serine protease activity, a requirement for *S. pneumoniae* killing after *P. melaninogenica* exposure, is similarly low in neutrophils of nonprimed and *E. coli* LPS-treated mice (20). Therefore, PBS treatment was used as a nonprimed, negative control for neutrophil functional assays moving forward. We used an in vivo flow cytometry-based uptake assay to assess the ability of lung neutrophils to phagocytose *S. pneumoniae*. Neutrophil phagocytosis of *S. pneumoniae* was significantly increased in the lungs of *P. melaninogenica*-primed mice (Fig. 3*B*). In addition to phagocytic and killing capacity, neutrophils were analyzed for surface expression of CD18, an integrin subunit which together with CD11b forms complement receptor 3 (CR3), important for phagocytosis of iC3b-opsonized bacteria (26). Neutrophil expression of CD18 was significantly higher in *P. melaninogenica*-primed mice (Fig. 3*C* and *SI Appendix*, Fig. S5*A*), correlating with the increased phagocytosis and *S. pneumoniae* killing. Of note, C3 and other complement associated genes were upregulated in neutrophils and macrophages in *P. melaninogenica*-primed mice (*SI Appendix*, Fig. S5*B*).

Unbiased analysis of the two neutrophil populations identified by scRNA-seq revealed significant enrichment for several gene ontology (GO) terms involved in immune defense in *P. melaninogenica*-exposed mice (Fig. 3*D*). To investigate differences in phagocytosis and toll-like receptor signaling associated genes, a module scoring function was applied to assess expression of selected gene sets in aggregate per condition. Module scoring analysis identified a significant upregulation of both phagocytosis signature genes and toll-like receptor signaling genes (Fig. 3*E*), which stimulate neutrophil activation, phagocytosis, and reactive oxygen species production (27), in *P. melaninogenica*-exposed mice. Module scoring also indicated increased maturation associated genes in the Mmp9+ neutrophil population, which was smaller in total number compared to the largely expanded Csf1+ neutrophil population, and significantly elevated migration signature genes in both neutrophil populations in *P. melaninogenica*-exposed mice (*SI Appendix*, Fig. S5*C*). Several neutrophil granule genes were reduced in the Csf1+ neutrophil population of *P. melaninogenica*-primed mice (Fig. 3*F*), which may indicate regulation of granule synthesis in more matured neutrophils with sufficiently accumulated granules (28, 29). These findings suggest that *P. melaninogenica* upregulates antibacterial defense genes in lung neutrophils.

### TNF Signaling Drives Enhanced Neutrophil-Mediated Clearance of *S. pneumoniae* Following *P. melaninogenica* Priming.

**Fig. 4.**
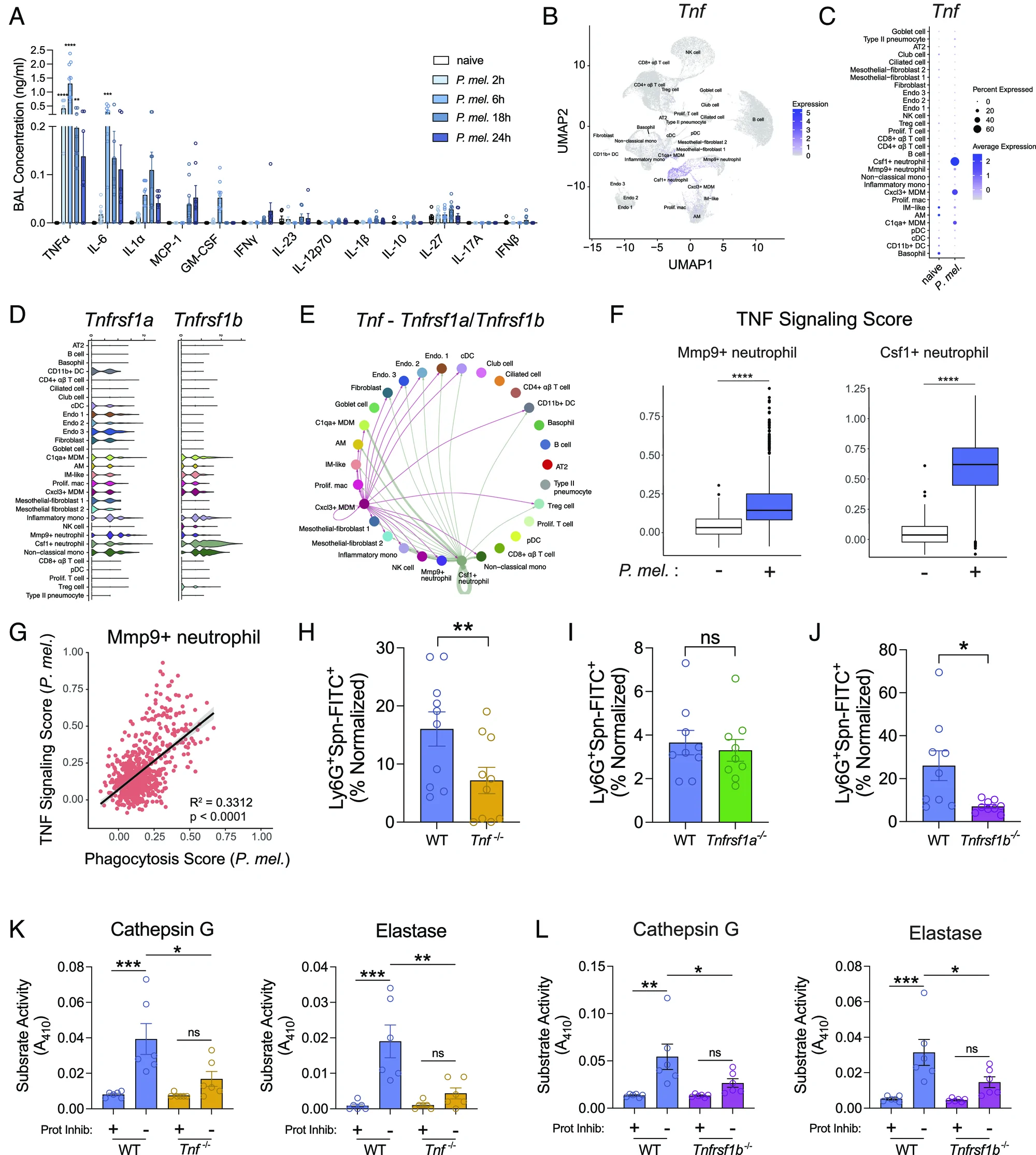
TNF signaling drives enhanced neutrophil-mediated clearance of *S. pneumoniae* following *P. melaninogenica* priming. (*A*) Quantification of cytokines and chemokines in lung BAL of mice exposed to *P. mel* HK (mean ± SEM; n = 7-9 mice/group from three independent experiments). (*B*) UMAP overlay of *Tnf* gene expression. (*C*) Dot plot of *Tnf* expression in naïve vs. *P. mel* HK-exposed mice. (*D*) Violin plot of *Tnfrsf1a* and *Tnfrsf1b* expression across all cell populations. (*E*) CellChat circle plot displaying inferred *Tnf*-*Tnfrsf1a*/*Tnfrsf1b* signaling network. (*F*) Module scoring analysis of TNF signaling signature genes in neutrophils of naïve mice and mice exposed to *P. mel* HK. (*G*) Pearson’s correlation of phagocytosis and TNF signaling module scores for the Csf1+ neutrophil population from *P. mel* HK-exposed mice. Quantification of Ly6G+Spn-FITC+ neutrophils isolated from WT vs. *Tnf*^−/−^ (*H*) *Tnfrsf1a*^−/−^ (*I*), or *Tnfrsf1b*^−/−^ mice (*J*) and incubated with FITC-labeled *Spn* HK for 30 min (mean ± SEM; n = 9-10 mice/group from three to four independent experiments). Serine protease activity detected in neutrophils purified from WT vs. *Tnf*^−/−^ (*K*) or *Tnfrsf1b*^−/−^ (*L*) mice, +/− protease inhibitor cocktail (mean ± SEM; n = 6 mice/group from two independent experiments). Data were analyzed by two-way ANOVA with Dunnett’s post hoc test (*A*), Mann–Whitney test (*H* and *I*), unpaired *t* test (*J*), or ordinary one-way ANOVA with Šídák’s post hoc test (*K* and *L*). *P < 0.05, **P < 0.01, ***P < 0.001, ****P < 0.0001.

In BAL fluid from mice exposed to HK *P. melaninogenica*, TNFα was the highest measured cytokine, produced as early as 2 h postexposure to *P. melaninogenica* (Fig. 4*A*). Neutrophils were the primary *Tnf* expressing cells, with expression also noted in monocyte and macrophage populations, and *Tnf* expression was elevated in *P. melaninogenica*-exposed mice (Fig. 4 *B* and *C*). TNFα is recognized by TNF receptor 1 (TNFR1) and TNFR2, which are encoded by the *Tnfrsf1a* and *Tnfrsf1b* genes, respectively. As expected, *Tnfrsf1a* was broadly expressed across different lung cell types, while *Tnfrsf1b* was primarily expressed by myeloid cells and T regulatory cells (30) (Fig. 4*D*). Both TNF receptors were highly expressed by neutrophils. Using CellChat analysis to infer cell–cell interactions, neutrophils were predicted to respond to TNFα produced by Cxcl3+ MDMs and from themselves in an autocrine fashion (Fig. 4*E*). By module scoring analysis, both neutrophil populations in *P. melaninogenica*-exposed mice exhibited significant upregulation of TNF signaling pathway associated genes (Fig. 4*F*). Linear correlation demonstrated a significant positive correlation between expression of phagocytosis-related and TNF signaling pathway genes in the Mmp9+ neutrophil population of *P. melaninogenica*-exposed mice (Fig. 4*G*).

To test whether TNFα signaling regulates *S. pneumoniae* phagocytosis, neutrophils were isolated from the lungs of wildtype and *Tnf*^−/−^ mice 24 h after *P. melaninogenica* exposure and incubated with pre-opsonized, FITC-labeled HK *S. pneumoniae* using an ex vivo uptake assay. In the absence of TNFα, neutrophil phagocytosis of *S. pneumoniae* was significantly reduced (Fig. 4*H*). To determine which TNFα receptors were necessary for enhanced *S. pneumoniae* phagocytosis, uptake of FITC-labeled HK *S. pneumoniae* was evaluated in neutrophils purified from wildtype, *Tnfrsf1a^−/−^*, or *Tnfrsf1b^−/−^* mice following *P. melaninogenica* priming. While phagocytic capacity was retained in neutrophils lacking TNFR1, *S. pneumoniae* phagocytosis was significantly reduced in neutrophils lacking TNFR2 compared to neutrophils from WT mice (Fig. 4 *I* and *J*). Neutrophils lacking either TNF or TNFR2 also exhibited significantly reduced serine protease activity (Fig. 4 *K* and *L*). Neutrophil expression of reactive oxygen species, which also contributes to intracellular bacterial killing, was unaffected by the absence of TNFα (*SI Appendix*, Fig. S5*D*). Loss of TNFα or TNFR2 did not impact neutrophil frequencies in naïve or *P. melaninogenica*-exposed mice (*SI Appendix*, Fig. S5 *E* and *F*). These data identify TNFα signaling through TNFR2 as a previously unrecognized regulator of neutrophil serine protease activity and bacterial uptake in the lung during acute pneumococcal pneumonia.

### Protective Immune Priming Reduces the Pneumococcal-Induced Type I IFN Response.

**Fig. 5.**
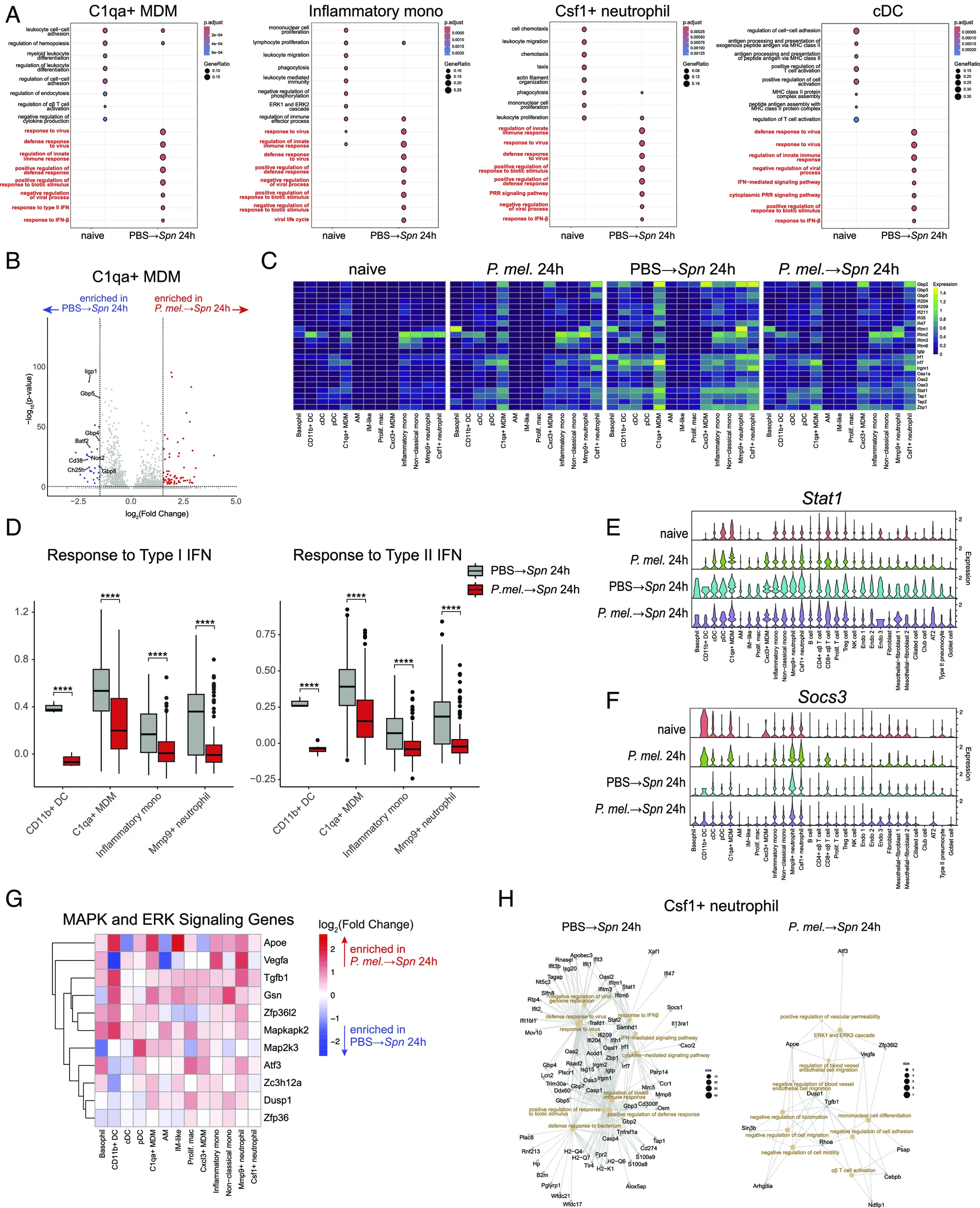
Protective immune priming reduces the pneumococcal-induced IFN response. (*A*) Top enriched GO pathways in selected myeloid cell populations from naïve vs. Spn-infected mice. Red text indicates GO Terms in which enriched genes are predominantly ISGs. (*B*) Volcano plot of top enriched genes in C1qa+ MDMs from *Spn*-infected mice with vs. without prior *P. mel* HK exposure, with individual ISGs indicated. (*C*) Heatmap of ISG expression across myeloid cell populations. (*D*) Module scoring analysis of type I interferon and type II interferon signaling signature genes in myeloid cell populations of *Spn*-infected mice +/− *P. mel* HK. Violin plots showing expression of (*E*) *Stat1* and (*F*) *Socs3* across all cell types, by condition. (*G*) Heatmap displaying log-fold changes of MAPK signaling gene expression in myeloid cells of mice exposed to *P. mel* HK prior to *Spn* infection, relative to mice infected with *Spn* alone. (*H*) Network plot of top enriched pathways (beige nodes) and associated genes in the Csf1+ neutrophil population for *Spn*-infected mice +/− *P. mel* HK exposure. Data were analyzed by Wilcoxon’s rank-sum test (*D*). ****P < 0.0001.

In mice infected with *S. pneumoniae* alone, we identified expression of several genes associated with canonical innate immune activation during pneumococcal infection (*SI Appendix*, Fig. S6*A*). Using pathway enrichment analysis as an unbiased approach to identify the top differentially enriched signaling pathways, type I and type II interferon associated pathways emerged as the dominant gene ontology pathways across myeloid cell populations in *S. pneumoniae*–infected mice (Fig. 5*A*). Interferon-stimulated genes (ISGs) were also among the top upregulated genes in the C1qa+ MDM population which was selectively enriched in *S. pneumoniae* infected mice (Fig. 5*B*) and broadly among myeloid cells (Fig. 5*C*). Correspondingly, expression of type I interferon receptor genes *Ifnar1* and *Ifnar2*, as well as type II interferon receptor gene *Ifngr1*, were higher in myeloid populations compared to other cell types (*SI Appendix*, Fig. S6*B*). Notably, ISGs were expressed at lower levels in mice exposed to *P. melaninogenica* prior to *S. pneumoniae* infection (Fig. 5*C*), in which enhanced *S. pneumoniae* clearance was observed. While similar interferon-associated genes were upregulated during *S. pneumoniae* infection, particularly among C1qa+ MDMs and neutrophils, module scoring analysis indicated a significant reduction in the expression of both type I and type II interferon signature genes in these cells, as well as inflammatory monocytes and dendritic cells, in mice primed with *P. melaninogenica* prior to *S. pneumoniae* infection (Fig. 5*D*).

Expression of *Stat1*, which controls expression of interferon-stimulated genes (31), was upregulated to a greater degree in all cell types in the lungs of *S. pneumoniae*–infected mice compared to *P. melaninogenica*-exposed mice (Fig. 5*E*). In contrast, suppressor of cytokine signaling 3 (*Socs3*), a negative regulator of the JAK/STAT signaling pathway (32), was increased to a greater degree in myeloid cells and T cells of *P. melaninogenica*-exposed mice compared to *S. pneumoniae*–infected mice (Fig. 5*F*). Across myeloid cell populations, MAPK and ERK signaling associated genes were upregulated in mice primed with *P. melaninogenica* prior to *S. pneumoniae* infection, compared to mice infected with *S. pneumoniae* alone (Fig. 5*G*). Similarly, network pathway analysis revealed ERK1 and ERK2 cascade signaling as a top upregulated pathway in neutrophils from mice primed with *P. melaninogenica* before *S. pneumoniae* infection, compared to several interferon signaling related pathways dominating neutrophils in unprimed mice infected with *S. pneumoniae* (Fig. 5*H*). Of note, MAPK and ERK1/2 signaling occur downstream of TLR2 (33), shown to be required for *P. melaninogenica*-mediated protection (20). Additionally, genes involved in chemotaxis and phagocytosis, processes known to be regulated by MAPK-ERK signaling (34–36), were enriched in *P. melaninogenica*-primed mice (*SI Appendix*, Fig. S5*G*). These data suggest that the improved immune-mediated clearance of *S. pneumoniae* in mice exposed to *P. melaninogenica* involves an overall transcriptional shift away from an interferon signaling-dominant profile toward a pro-phagocytic profile associated with MAPK/ERK signaling.

### Transcriptional Changes in Monocytes and Macrophages Associated with Increased Phagocytic Capacity.

**Fig. 6.**
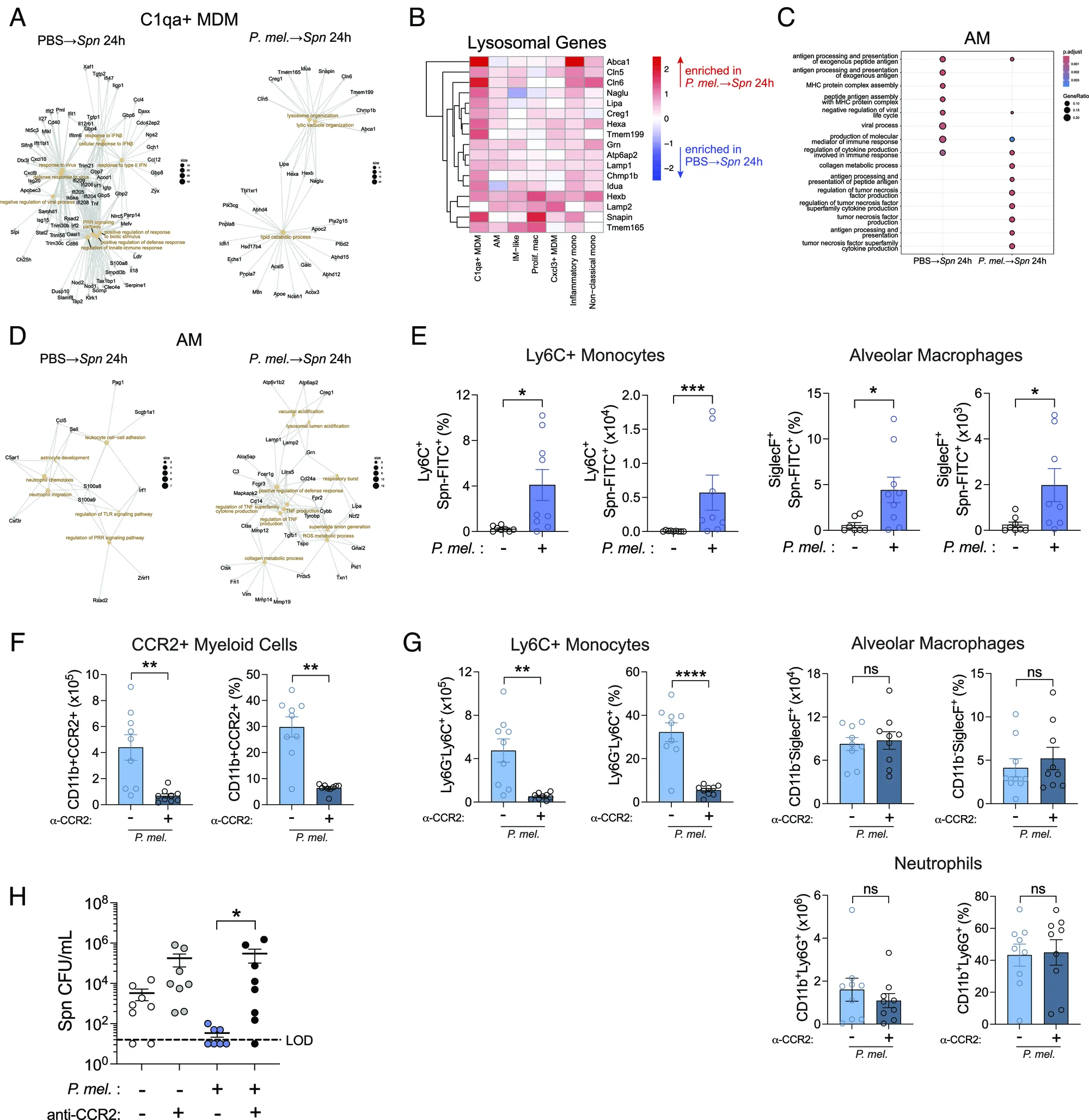
Monocyte and macrophage populations contribute to *P. melaninogenica*–induced clearance of *S. pneumoniae*. (*A*) Network plot of top enriched pathways (beige nodes) and associated genes in C1qa+ MDMs from *Spn*-infected mice +/− *P. mel* HK exposure. (*B*) Heatmap of log-fold changes in lysosomal gene expression in monocyte and macrophage populations from mice exposed to *P. mel* HK prior to *Spn* infection, relative to mice infected with *Spn* alone. (*C*) Top enriched GO pathways in AMs from *Spn*-infected mice +/− *P. mel* HK exposure. (*D*) Network plot of top enriched pathways and associated genes in AMs from *Spn*-infected mice +/− *P. mel* HK exposure. (*E*) Ly6C+Spn-FITC+ monocytes and SiglecF+Spn-FITC+ AMs in lung tissue of mice exposed to FITC-labeled *Spn* HK for 3 h, +/− *P. mel* HK (mean ± SEM; n = 7-9 mice/group from three independent experiments). (*F*) CCR2+ myeloid cells in mice treated with isotype control or anti-CCR2 (MC-21) antibodies i.p. prior to *P. mel* HK exposure (mean ± SEM; n = 9 mice/group from three independent experiments). (*G*) Ly6G-Ly6C+ monocytes, CD11b-SiglecF+ AMs, and CD11b+Ly6G+ neutrophils in mice treated as in (*F*), (mean ± SEM; n = 9 mice/group from three independent experiments). (*H*) Lung Spn burdens in mice treated with isotype control or anti-CCR2 antibodies together with PBS or *P. mel* HK exposure prior to *Spn* infection (1 × 10^7^ CFU) (mean ± SEM; n = 8-9 mice/group from three independent experiments). Data were analyzed by the Mann–Whitney test (*E*–*G*), unpaired *t* test (*E*–*G*), or Kruskal–Wallis with Dunn’s post hoc test (*H*). *P < 0.05, **P < 0.01, ***P < 0.001.

We next analyzed the transcriptional differences in macrophages from *P. melaninogenica* primed mice. In C1qa+ MDMs, lysosome and lytic vacuole organization genes were enriched in mice exposed to *P. melaninogenica* prior to *S. pneumoniae* infection, compared to mice infected with *S. pneumoniae* alone (Fig. 6*A*). Lysosome organization genes were broadly upregulated across all monocyte and macrophage populations in mice exposed to *P. melaninogenica* prior to *S. pneumoniae* infection (Fig. 6*B*). Similarly, unbiased pathway enrichment analysis identified several pathways associated with bacterial killing in AMs from mice exposed to *P. melaninogenica* before *S. pneumoniae* infection, including lysosomal acidification and reactive oxygen species metabolic process associated genes (Fig. 6*C*). Network pathway analysis confirmed enrichment in lysosome regulation and positive regulation of defense response genes in AMs for *P. melaninogenica*-primed, but not unprimed, mice infected with *S. pneumoniae* (Fig. 6*D*). Bacterial phagocytosis has been shown to increase expression of lysosomal genes in macrophages (37). To determine the functional correlates of the lysosome-associated transcriptional differences, we performed an in vivo phagocytosis assay to compare *S. pneumoniae* uptake in mice with or without *P. melaninogenica* exposure. Assessment of phagocytic capacity revealed a significant increase in phagocytosis of FITC-labeled HK *S. pneumoniae* for both recruited Ly6C+ monocytes and MDMs as well as resident AMs in mice primed with *P. melaninogenica* (Fig. 6*E*).

### Monocyte and Macrophage Populations Contribute to *P. melaninogenica*–Induced Clearance of *S. pneumoniae*.

Given their increased bacterial uptake, we sought to understand whether recruited monocytes and macrophages directly contribute to enhanced clearance of *S. pneumoniae* in *P. melaninogenica*-primed mice. To address this question, mice were treated with an anti-CCR2 antibody, which selectively depleted CCR2+ myeloid cells, leading to a significant reduction in Ly6C+ recruited monocytes and macrophages without affecting resident AMs or recruited neutrophil populations (Fig. 6 *F* and *G*). In the absence of recruited monocytes and macrophages, *P. melaninogenica* exposure was no longer protective against *S. pneumoniae* infection (Fig. 6*H*). Notably, neutrophil activation, as measured by CD18 surface expression and intracellular TNFα, was not affected by anti-CCR2 treatment (*SI Appendix*, Fig. S7 *A* and *B*), suggesting that regulation of neutrophil-mediated bacterial killing was not the mechanism responsible for CCR2-dependent enhanced protection against *S. pneumoniae* infection in *P. melaninogenica*-primed mice. Together, these data reveal a previously unrecognized dependence on recruited monocytes and macrophages for commensal-enhanced protection against pneumococcal pneumonia.

## Discussion

Using a preclinical model recapitulating associations between *Prevotella* aspiration and improved outcomes in critically ill patients, we demonstrate that *P. melaninogenica* dramatically alters the pulmonary transcriptional innate immune response to *S. pneumoniae*. The gene expression profile of *P. melaninogenica*-exposed mice in myeloid cells was enriched in antibacterial, pro-phagocytic pathways, in contrast to the antiviral, interferon-dominant expression profile observed in nonprimed mice with unresolved infection. Macrophages and monocytes of *Prevotella*-primed mice exhibited functional increases in phagocytic capacity and directly participated in *S. pneumoniae* clearance. Further, we identified an essential role for TNFR2 in neutrophil-mediated protection during pneumococcal pneumonia, mediated by increased neutrophil phagocytosis and serine protease activity. Together, these findings illustrate a paradigm for improved antibacterial defense through commensal-directed myeloid cell reprogramming.

In contrast to *P. melaninogenica* and other airway *Prevotella* species, we previously found that *Prevotella intermedia* was not protective and failed to induce a similar TLR2-dependent neutrophil TNFα response as the “protective” species (20). Consistent with this observation, others report species-specific TLR activation in epithelial cells, as *Prevotella histicola* activated TLR2 and TLR5, while *Prevotella nigrescens* selectively activated TLR2 (38, 39). Unlike *P. melaninogenica*, *P. intermedia* is frequently associated with periodontal abscesses, harbors several virulence factors, and upregulates host adherence-associated receptor *Ptafr*, correlating with increased *S. pneumoniae* burdens (40, 41). Here, we focused on defining the transcriptional reprogramming induced by exposure to *P. melaninogenica* to uncover the transcriptional changes associated with improved *S. pneumoniae* clearance, compared to less robust infection defense.

To our surprise, the dominant transcriptional signature enriched in myeloid cells during *S. pneumoniae* infection without *Prevotella* priming was an antiviral associated interferon response. *S. pneumoniae* triggers type I interferon signaling via the pore-forming toxin pneumolysin (42, 43). Interferon signaling can protect against *S. pneumoniae* infection by increasing alveolar epithelial cell survival and myeloid cell activation (44, 45) and *Ifnar*^−/−^ mice exhibit increased *S. pneumoniae* burdens (42, 46), suggesting that the complete absence of type I interferon signaling is harmful. However, excessive type I interferon signaling is widely documented to increase susceptibility to pneumococcal pneumonia and worsen pneumonia outcomes, particularly in the context of viral coinfections (47–50), and type I interferon can negatively regulate phagocytosis (44, 48, 49). These findings suggest that the magnitude of the type I interferon response may determine whether it is beneficial or detrimental to *S. pneumoniae* clearance. In myeloid cells from *P. melaninogenica* primed mice, reduced overall ISG expression corresponded with elevated expression of MAPK and ERK signaling genes, aligning functionally with increased *S. pneumoniae* uptake by lung neutrophils and macrophages in *P. melaninogenica*-primed mice. TLR2 and MAPK signaling are associated with enhanced phagocytosis and pathogen killing by innate immune cells in other infection models (51, 52). Together, these data suggest that an interferon dominant response is maladaptive for efficient *S. pneumoniae* clearance, compared to the TLR2 and MAPK/ERK signaling program induced by *Prevotella* priming.

The recruitment of monocyte-derived macrophages to the lungs is a hallmark of severe pneumonia, with direct contributions to alveolar injury (53–55). We observed striking differences among the two transcriptionally distinct MDM populations identified by scRNA-seq between mice infected with *S. pneumoniae* compared to those primed with *P. melaninogenica*. The C1qa+ MDM population which dominated *S. pneumoniae*–infected mice expressed significantly higher ISGs and may therefore serve as a key source of the maladaptive interferon response observed in susceptible mice. In people with severe bacterial pneumonia, recent reports indicate a central role for circulating C1qa-expressing monocytes in driving deleterious inflammation (56, 57). In contrast, the Cxcl3+ MDM population selectively enriched by *P. melaninogenica* exposure transcriptionally resembled mature tissue resident-like macrophages and expressed chemokines that contribute to neutrophil recruitment, suggesting these two populations have distinct impacts on the immune landscape during infection. Other examples of enhanced defense against bacterial pneumonia following immune priming by microbial-derived factors, gut commensals, or other pneumococcal serotypes are all mechanistically linked to reprogramming in resident macrophages, namely AMs (58–62). Here, our findings provide insight regarding how distinct MDM subpopulations recruited during infection can alter the protective host response.

Neutrophils undergo several maturation states following tissue migration and during bacterial infection (63), with two dominant populations frequently reported (64, 65). Neutrophil cell numbers were lower in *S. pneumoniae*–infected mice after *P. melaninogenica* priming, consistent with lower pathogen burdens and a resolving inflammatory response. Of the two neutrophil populations we identified, Csf1+ neutrophils expressed higher levels of inflammatory genes, including those involved in TLR signaling, phagocytosis, and TNF signaling, in response to *P. melaninogenica*. The independent contributions of TNFR1 vs. TNFR2 signaling during infection are largely unclear. Here, we demonstrate that TNFR2 is required for enhanced phagocytosis and serine protease production in neutrophils after *P. melaninogenica* priming. A protective role for TNFR2 has previously been found during skin infection with *S. aureus*, where TNFR2 promoted activation of neutrophil ROS and vital NETosis (66). In contrast, we found that TNFR2 signaling increased neutrophil phagocytosis and serine protease activity without altered ROS, suggesting that the protective functions of TNFR2 signaling are context-dependent. While *P. melaninogenica* exposure can directly induce neutrophil production of TNF⍺ ex vivo, additional tissue-specific cells or signals are required for enhanced *S. pneumoniae* killing (20). Here, we cannot rule out the contribution of TNFR2 expression on other cells for enhanced neutrophil antimicrobial function. Together, these findings highlight TNF signaling through TNFR2 as a critical regulator of neutrophil serine protease activity, which is a key mechanism of *S. pneumoniae* killing (67, 68).

This study has several limitations to consider. Many immune defense mechanisms are regulated posttranscriptionally; therefore, gene expression data were complemented with protein analysis and functional assays whenever possible. While single dose pre-exposure models are commonly used to delineate immune priming mechanisms (19, 69–71), future work could expand these findings using multiple smaller doses over an extended period, mimicking continuous exposure to aspirated oral commensals. Another limitation is the use of HK bacteria, as recent work demonstrates that lower airway enrichment of oral commensals correlates with the detection of immune-modulating short-chain fatty acids (72), and that *P. melaninogenica* is metabolically active in the murine lung (72). These findings provide initial evidence that aspirated oral commensals are not merely transient passengers but active participants shaping the lung immune environment.

Clinically, escalating evidence suggests that airway anaerobes including *Prevotella* should be spared during treatment of critically ill patients, based on the strong associations between anaerobes and improved survival (9, 73–75). While arguments for this approach are often centered on the potential contributions of gut anaerobes, a recent study concluded that oral and lung anaerobes are the most powerful microbial indicator of improved survival in critically ill patients (76). In the same study, *Prevotella* inversely correlated with acute respiratory failure and markers of tissue injury including soluble TNFR1 (76). This observation is in alignment with the protective TNF signaling through TNFR2, rather than pro-apoptotic TNFR1, observed in *P. melaninogenica* exposed mice. Our data, together with the clinical correlation between *P. melaninogenica* abundance and reduced lower airway infections (8, 11, 15), point to an important role for *P. melaninogenica* aspiration in directing lung immune homeostasis and defense against bacterial pneumonia. Further work is needed to optimize *Prevotella*-mediated enhanced defense in a clinical setting, with the potential to reduce overall pneumonia mortality.

## Materials and Methods

C57BL/6J (WT), B6.129S-Tnf ^tm1Gkl^/J (*Tnf*^−/−^), C57BL/6-Tnfrsf1a^tm1lmx^/J (*Tnfrsf1a*^−/−^), and B6.129S2-Tnfrsf1b^tm1Mwm^/J (*Tnfrsf1b*^−/−^) mice were purchased from The Jackson Laboratory. For infections, mice were administered 10^7^ CFU *S. pneumoniae* i.t. in 50 μL PBS. For *P. melaninogenica* exposures, mice were administered 50 μL i.t. 10^6^-10^8^ CFU equivalents of heat-killed (HK) bacteria in PBS 24 h prior to live *S. pneumoniae* infection. For CCR2+ monocyte depletion, mice were intraperitoneally injected with either 20 μg MC-21 antibody (77) or rat IgG2B isotype control (Bio X Cell) in 200 μL PBS 24 h prior and immediately before treating with HK *P. melaninogenica* i.t. Depletion of CD11b+CCR2+ myeloid cells was confirmed via flow cytometry. Detailed descriptions of remaining materials and methodology including bacterial strains and preparations, flow cytometry, cytokine and chemokine analysis, functional assays, single cell RNA sequencing, and data analysis, is provided in *SI Appendix*, *SI Materials and Methods* section.

## Supplementary Material

Appendix 01 (PDF)

## Data Availability

Transcriptomic data can be accessed in the NCBI Gene Expression Omnibus (GEO) under accession number GSE313404 (78). All other data are included in the manuscript and/or *SI Appendix*.
