# Supplementary material for: Myeloid cell reprogramming drives enhanced defense against Streptococcus pneumoniae lung infection following exposure to commensal Prevotella
Source: Proc Natl Acad Sci U S A. 2026 Oct 7;123(41):e2623525123. doi: 10.1073/pnas.2623525123 (PMC13663786; doi:10.1073/pnas.2623525123)
Supplement: Supplementary file 1 [file pnas.2623525123.sapp.pdf]

Supporting Information

SI Materials and Methods
SI References
SI Figures S1-S7

SI Materials and Methods

Study Approval

These studies were approved by the Animal Care and Use Committee of the University of Colorado School of Medicine (protocol #00927) and by the Institutional Biosafety Committee (protocol #1418).

Animals

Adult male and female mice aged 7-9 weeks were used for this study. Strains from The Jackson Laboratory included C57BL/6 J (WT, #000664), B6.129S-Tnf ^{tm1Gkl}/J (*Tnf*$^{-/-}$, #005540), C57BL/6-Tnfrsf1a^{tm1Imx}/J (*Tnfrsf1a*$^{-/-}$, #003242), and B6.129S2-Tnfrsf1b^{tm1Mwm}/J (*Tnfrsf1b*$^{-/-}$, #002620). All mouse strains used in these studies (WT, *Tnf*$^{-/-}$, *Tnfrsf1a*$^{-/-}$, and *Tnfrsf1b*$^{-/-}$) are on the C57BL/6J genetic background. Mice were maintained in the University of Colorado Office of Laboratory Animal Resources.

Bacteria

Prevotella melaninogenica strain ATCC® 25845 was purchased from the American Type Culture Collection (Manassas, VA). To prepare heat-killed *Prevotella* stocks, *P. melaninogenica* was grown on Brucella agar plates anaerobically for 2 days, then resuspended in PBS and incubated at 65°C for 30 min. A streptomycin resistant variant of serotype 2 *Streptococcus pneumoniae* strain D39 was used for these studies[1]. *S. pneumoniae* was grown statically in Todd Hewitt Broth with 5% Yeast Extract (BD Bacto™), with 50 µg/mL streptomycin (Sigma), at 37 °C with 5% CO_2. Heat-killed *S.*

pneumoniae was prepared by growing to OD_{600} > 0.5, resuspending in PBS, and incubating at 65°C for 1 hour. Samples before and after heat-killing were used to determine CFU equivalents/mL and confirm killing, respectively. To prepare FITC-labeled *S. pneumoniae*, heat-killed bacteria were pelleted and resuspended in a 0.5mg/ml FITC solution in PBS and incubated for 1 hour at 4°C. HK *S. pneumoniae* was washed twice with Hank's Balanced Salt Solution (Gibco) + 1% bovine serum albumin (Fisher BioReagents™) and then resuspended in HBSS + 1% BSA.

<u>Infections</u>

For *S. pneumoniae* infections, bacteria were grown to mid-log phase in Todd Hewitt Broth with 5% Yeast Extract, centrifuged at ≥20,000 x *g* for 10 min, and resuspended in PBS before storing at -80°C. CFU plates were used to determine concentration of frozen stocks. For infections, *S. pneumoniae* frozen stocks were thawed and resuspended at desired concentration, and mice were administered 10^7 CFU *S. pneumoniae* i.t. in 50 µL PBS. For *P. melaninogenica* exposures, mice were administered 50 µL i.t. of 10^6-10^8 CFU in PBS 24 hours prior to *S. pneumoniae* infection. For CFU enumeration, lung tissue was homogenized using the Bullet Blender tissue homogenizer (Stellar Scientific). Lung homogenates were serially diluted and plated on blood agar plates (Remel).

<u>Flow Cytometry</u>

Lungs were harvested following perfusion by transcardial injection of 10 mL PBS, and single cells were prepared for flow cytometry [2]. Lung tissue was minced and then incubated in enzymatic digestion buffer (DNaseI 30 μg/mL, Sigma, and type 4 collagenase 1 mg/mL, Worthington Biochemical Corporation, in Hank's Balanced Salt Solution with calcium and magnesium) for 20 min at 37°C with 5% CO_2. Tissue was strained through a 70 μM cell strainer (Falcon). To remove red blood cells, samples were pelleted and resuspended in 1mL RBC lysis buffer (0.15 M NH_4Cl, 10 mM $KHC0_3$, 0.1 mM Na_2EDTA, pH 7.4) for 2 min. Fc receptors were blocked by incubation in anti-CD16/32 (BioLegend) prior to staining in FACS buffer (1% BSA, 0.01% NaN3, PBS). For reactive oxygen species experiments, cells were incubated with Dihydrorhodamine 123 (Sigma) for 20 min at 37°C prior to staining. For intracellular staining, samples were incubated with Brefeldin A (BD Biosciences) diluted 1:1000 for 3 hours at 37°C with 5% CO_2. Samples were permeabilized with 1 mg/mL saponin (Sigma) in FACS buffer prior to intracellular staining. Antibodies used for staining included anti-mouse: CD45.2 (BioLegend, catalog #564616, clone 104, lot #4339788), CD18 (BD, catalog #749471, clone M1 8/2, lot #5084986), Siglec F (BD, catalog #562681, clone E50-2440, lot #0269846), Ly6C (BioLegend, catalog #128033, clone HK1.4, lot #B432613), Ly6G (BioLegend, catalog #127614, clone 1A8, lot #B366717), CD11b (BioLegend, catalog #101226, clone M1/70, lot #B445235), and TNFα (Invitrogen, catalog #25-7321-82, clone MP6-XT22, lot #2250799). All cells were fixed in 1% paraformaldehyde for 20 min at room temperature. Flow cytometry was performed on the LSR Fortessa X-20 and the Cytek Aurora in the Cancer Center Flow Cytometry Shared Resource Laboratory at the University of Colorado Anschutz Medical Campus (RRID: SCR_022035). Analysis of

flow cytometry data was performed using FlowJo™ Software, version 10.10.0 (BD Life Sciences).

Cytokine and chemokine analysis

BAL cytokines and chemokines except for CXCL1, CXCL2, and CXCL5 were quantified via the LEGENDplex™ Mouse Inflammation Panel (BioLegend) and the LEGENDplex™ Mouse Chemokine Panel (BioLegend). Analytes were measured on the LSR Fortessa X-20 in the Cancer Center Flow Cytometry Shared Resource Laboratory at the University of Colorado Anschutz Medical Campus (RRID: SCR_022035). Data were analyzed using the LEGENDplex™ Data Analysis Software Suite (BioLegend). BAL CXCL1, CXCL2, and CXCL5 were measured using the Mouse CXCL1/KC, Mouse CXCL2/MIP-2, and Mouse CXCL5/LIX DuoSet ELISA kits (R&D Systems), respectively. Analytes were measured on a Synergy™ HT Microplate Reader (BioTek), and data were analyzed via GraphPad Prism.

Neutrophil Functional Assays

For ex vivo opsonophagocytic assays, neutrophils were isolated from the lungs of WT, *Tnf*$^{-/-}$, *Tnfrsf1a*$^{-/-}$, or *Tnfrsf1b*$^{-/-}$ mice following 24hr treatment with HK *P. melaninogenica* i.t., as indicated. Ly6G+ neutrophils were isolated via magnetic bead positive selection (MojoSort PE positive selection kit, BioLegend). In prior work, similar results were observed using positive or negative selection to isolate lung neutrophils [2]. For opsonization, 2x10^6 CFU of FITC-labeled HK *S. pneumoniae* were incubated with 7.6%

baby rabbit serum (MP Biomedicals) for 30 min at 37°C. HK *S. pneumoniae* was then incubated with 10^5 neutrophils in HBSS + 1% BSA for 30 min at 37°C while rotating, prior to flow cytometry staining. To measure phagocytosis, FITC+ neutrophils were quantified via flow cytometry using the LSR Fortessa X-20 in the Cancer Center Flow Cytometry Shared Resource Laboratory at the University of Colorado Anschutz Medical Campus (RRID: SCR_022035). For serine protease assays, neutrophils were isolated via positive selection from the lungs of WT, *Tnf*$^{-/-}$, or *Tnfrsf1b*$^{-/-}$ mice following 24-hour treatment with HK *P. melaninogenica* i.t., as indicated. 10^5 purified neutrophils were incubated with or without 1x HALT™ protease inhibitor cocktail (Thermo Fisher) for 30 min at 37°C while rotating. Cells were washed and lysed with 0.1% Triton X-100. Elastase substrate (0.85 mM MeOSuc-Ala-Ala-Pro-Val-*p*NA, Sigma) and cathepsin G substrate (0.1 mM Succinyl-Ala-Ala-Pro-Phe-pNA, Sigma) were added to neutrophil lysates and incubated for 45 min at 37°C in the dark. Substrate activity was measured by reading absorbance at 410nm on the Synergy™ HT Microplate Reader (BioTek) minus control wells with no neutrophils added.

<u>Fixed Single-cell RNA Sequencing</u>

Mouse lung tissue was minced and incubated in digestion buffer (DNaseI 0.4 mg/mL, Sigma, type 4 collagenase 1.5 mg/mL, Worthington Biochemical Corporation, 5% fetal bovine serum, CPS Serum, and 10mM HEPES Buffer, Gibco, in Hank's Balanced Salt Solution with calcium and magnesium) for 20 min at 37°C with 5% CO_2. Samples were then processed as described in the Flow Cytometry section. Cells with greater than 80%

viability were fixed in a 4% formaldehyde fixative solution using the 10X Genomics Chromium Next GEM Single Cell Fixed RNA Sample Preparation Kit. The whole transcriptome probe pairs (10X Genomics) were added to the fixed single cell suspensions to hybridize to their complementary target RNA in an overnight incubation at 42ºC. After hybridization, unbound probes were removed with a washing step. The fixed and probe-hybridized single cell suspensions were then loaded onto the Chromium X (10X Genomics) to generate partitioned nanoliter-scale droplets in oil emulsion, with each droplet containing a barcoded bead gel, a single cell, and enzyme Master Mix (10X Genomics) for probe pair ligation and gel bead primer barcode extension. The droplets in oil emulsion were placed in a thermal cycler for 60 min at 25ºC, 45 min at 60ºC, and 20 min at 80ºC. The single cell-barcoded, ligated probe products underwent library preparation using standard 10X Genomics protocols to ensure compatibility with Illumina next-generation sequencing. The gene expression library derived from single cell-barcoded, ligated probe products were sequenced as paired-end 150-base pair reads on the Illumina NOVASeq6000 (Illumina) at the University of Colorado Genomics Shared Resource core, Aurora, CO, USA (RRID: SCR_021984) at a depth of 20,000 reads per cell (1.34B reads total).

Data Analysis

FASTQ files were processed using CellRanger *multi* along with the Chromium Mouse Transcriptome Probe Set (v1.0.1 mm10-2020-A). Per-sample output feature matrices were used as the input to Seurat (v5) for further analysis. Count matrices were exported for analysis by Scrublet[3] to infer potential doublets. Scrublet output were added as

metadata to the Seurat object. Low quality and potential doublet cells were filtered using parameters: nCount RNA > 500 or <10000, nFeature_RNA >250, percent.mt < 15, doublet score <= 90th percentile. Dataset normalization and scaling was performed using 'SCTransform' and was visualized by PCA and UMAP (using 30 dimensions). Integration across conditions was performed using the RPCA method. Nearest-neighbor graph was constructed using 30 dimensions, the integrated reduction, and k =30. Initial clustering was performed using a resolution of 0.2. Based on cluster markers, cells were classified as myeloid, stromal, endothelial, epithelial, or lymphocyte. Each subset was then further subclustered at a resolution of 0.1 (except myeloid lymphocytes at 0.2). Cell identities were assigned using a combination of module scoring with known canonical cell type markers, cell type annotation tools (Annotation of Cell Types [4]; input as top 30 cluster markers per cluster), and manual identification.

Trajectory inference analysis of the macrophage subset was performed using PAGA [5]. An AnnData object was created from the Seurat object while retaining cell identities, PCs, and variable features. Gene set enrichment analysis and over representation analysis was performed using the GO:ALL database of ClusterProfiler [6] and significant cluster markers or DEGs between conditions as input. Cell communication pathways were inferred using CellChat v2 [7]. Module scoring for specific pathways was calculated using the 'AddModuleScore' function of Seurat. Custom gene lists used for modules are included in Source Data. For the Neutrophil module correlation, module scores were plotted and fit with a linear model. For all analyses, default parameters were used unless otherwise specified.

Statistical Analysis

For biological experiments, statistical analyses were performed using GraphPad Prism. Significant outliers were detected using the ROUT method (Q = 1%) and removed. All data were tested for normality via the Shapiro-Wilk test. For normally distributed data, unpaired t test, one-way ANOVA with Dunnett's or Šídák's post-hoc analyses, or two-way ANOVA with Dunnett's post-hoc analysis were used as specified. For non-Gaussian distributed data, Mann-Whitney tests or Kruskal-Wallis tests with Dunn's post-hoc analysis were used. For all statistical tests, p-values < 0.05 were considered significant.

SI References

1. Zafar, M. A., Kono, M., Wang, Y., Zangari, T. & Weiser, J. N. Infant Mouse Model for the Study of Shedding and Transmission during Streptococcus pneumoniae Monoinfection. *Infect Immun* **84**, 2714–2722 (2016).
2. Horn, K. J., Schopper, M. A., Drigot, Z. G. & Clark, S. E. Airway Prevotella promote TLR2-dependent neutrophil activation and rapid clearance of Streptococcus pneumoniae from the lung. *Nat Commun* **13**, 3321 (2022).
3. Wolock, S. L., Lopez, R. & Klein, A. M. Scrublet: Computational Identification of Cell Doublets in Single-Cell Transcriptomic Data. *Cell Syst* **8**, 281-291.e9 (2019).
4. Quan, F. *et al.* Annotation of cell types (ACT): a convenient web server for cell type annotation. *Genome Med* **15**, 91 (2023).

5. Wolf, F. A. *et al.* PAGA: graph abstraction reconciles clustering with trajectory inference through a topology preserving map of single cells. *Genome Biol* **20**, 59 (2019).
6. Wu, T. *et al.* clusterProfiler 4.0: A universal enrichment tool for interpreting omics data. *Innovation (Camb)* **2**, 100141 (2021).
7. Jin, S., Plikus, M. V. & Nie, Q. CellChat for systematic analysis of cell-cell communication from single-cell transcriptomics. *Nat Protoc* **20**, 180–219 (2025).

SI Figures

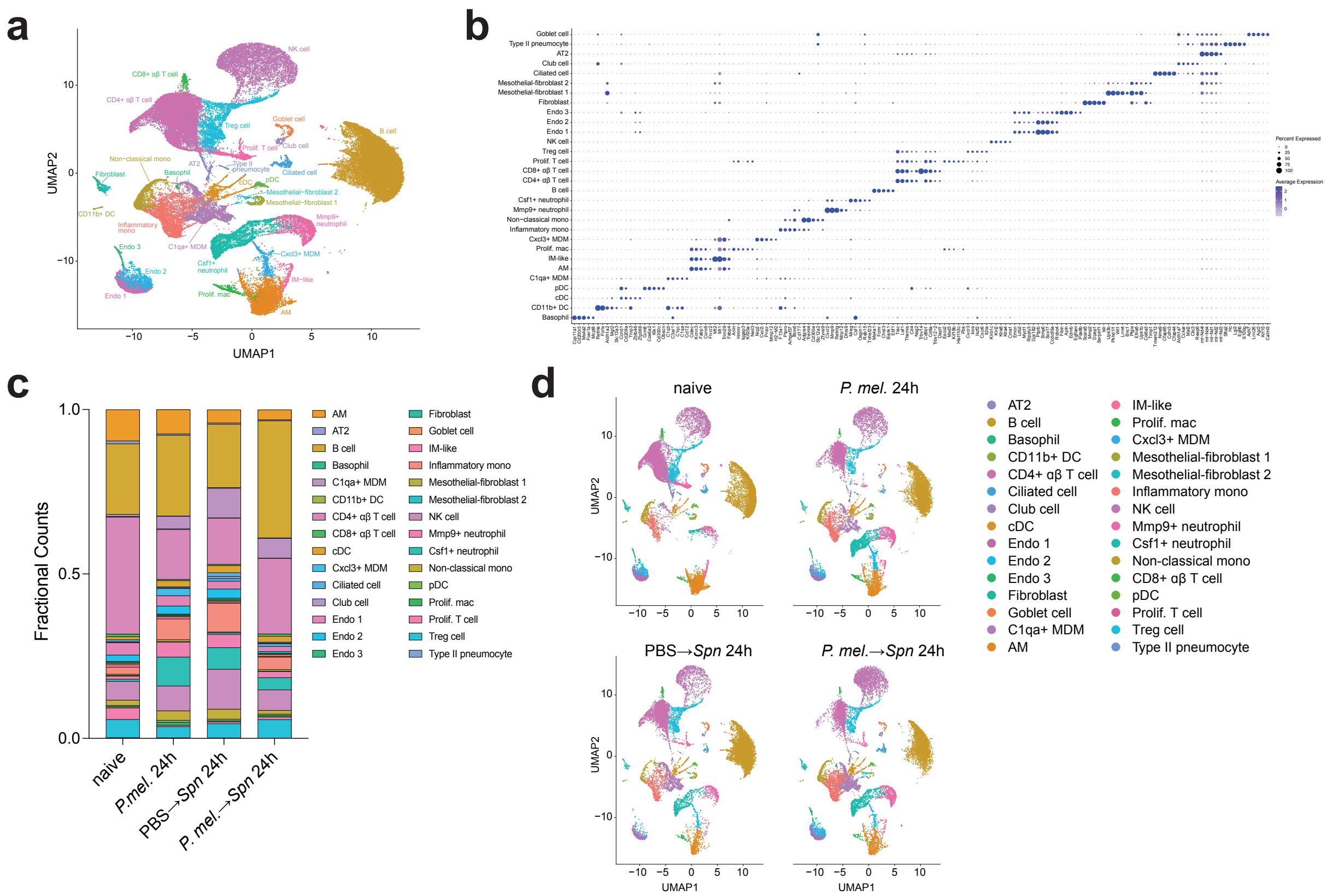

Figure S1: Landscape of lung tissue cellular response to *P. melaninogenica* and *Spn* infection detected by scRNA-seq.

(**A**) UMAP projection displaying all lung tissue cell populations identified by scRNA-seq. (**B**) Top 5 DEGs for all cell populations. (**C**) Fractional counts for all cell populations split by condition. (**D**) UMAP projections of all cell populations split by condition. Abbreviations: AT2 = alveolar type-2, endo = endothelial, NK = natural killer, Treg = T regulatory cell, Prolif = proliferating, mono = monocyte, MDM = monocyte-derived macrophage, IM = interstitial macrophage, mac = macrophage, AM = alveolar macrophage, DC = dendritic cell, pDC = plasmacytoid DC, cDC = conventional DC.

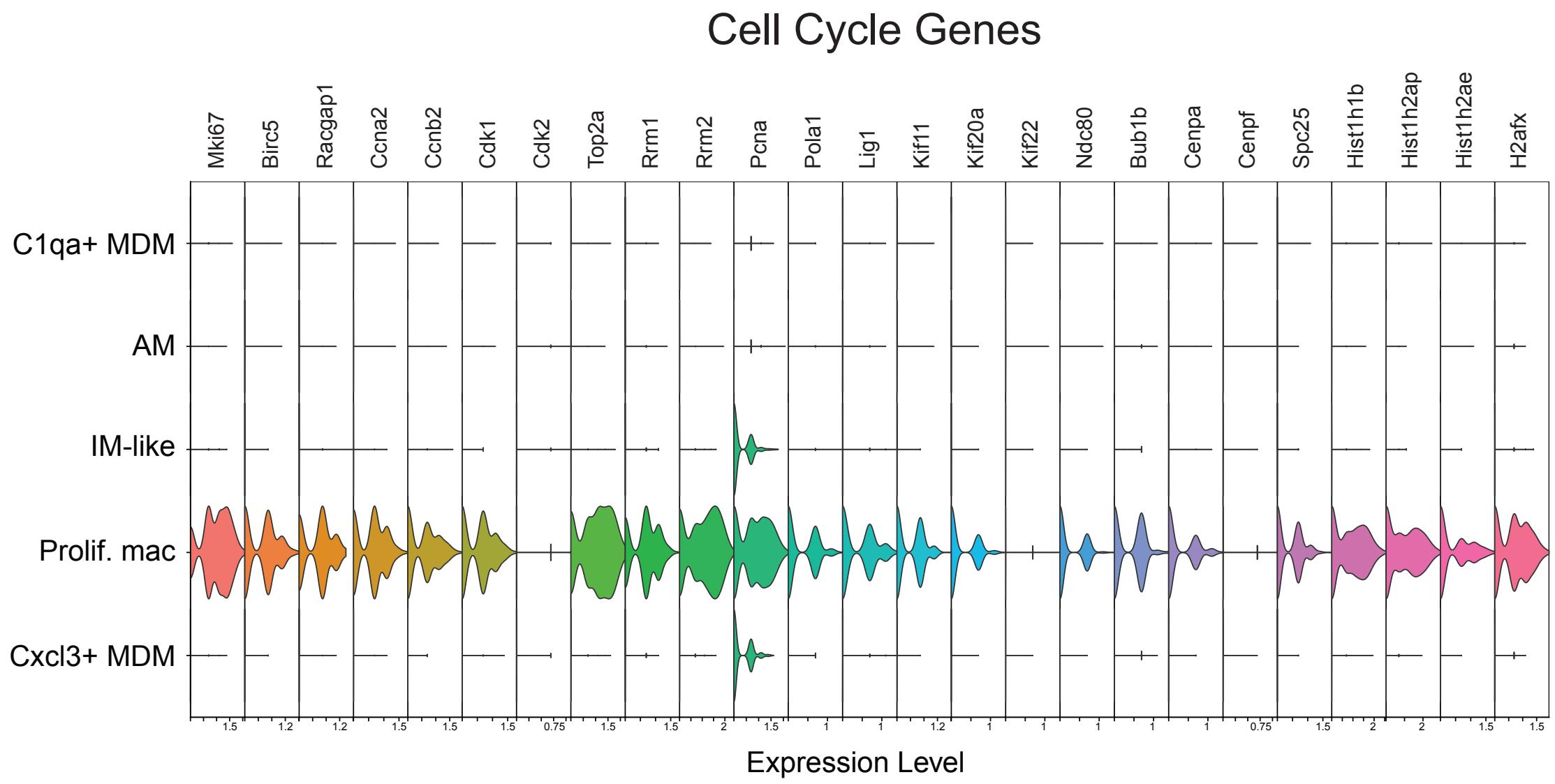

Figure S2: Expression of cell cycle genes in macrophages.

Violin plot showing expression of 25 cell cycle genes across macrophage populations. Abbreviations: Prolif = proliferating, mono = monocyte, MDM = monocyte-derived macrophage, IM = interstitial macrophage, mac = macrophage, AM = alveolar macrophage.

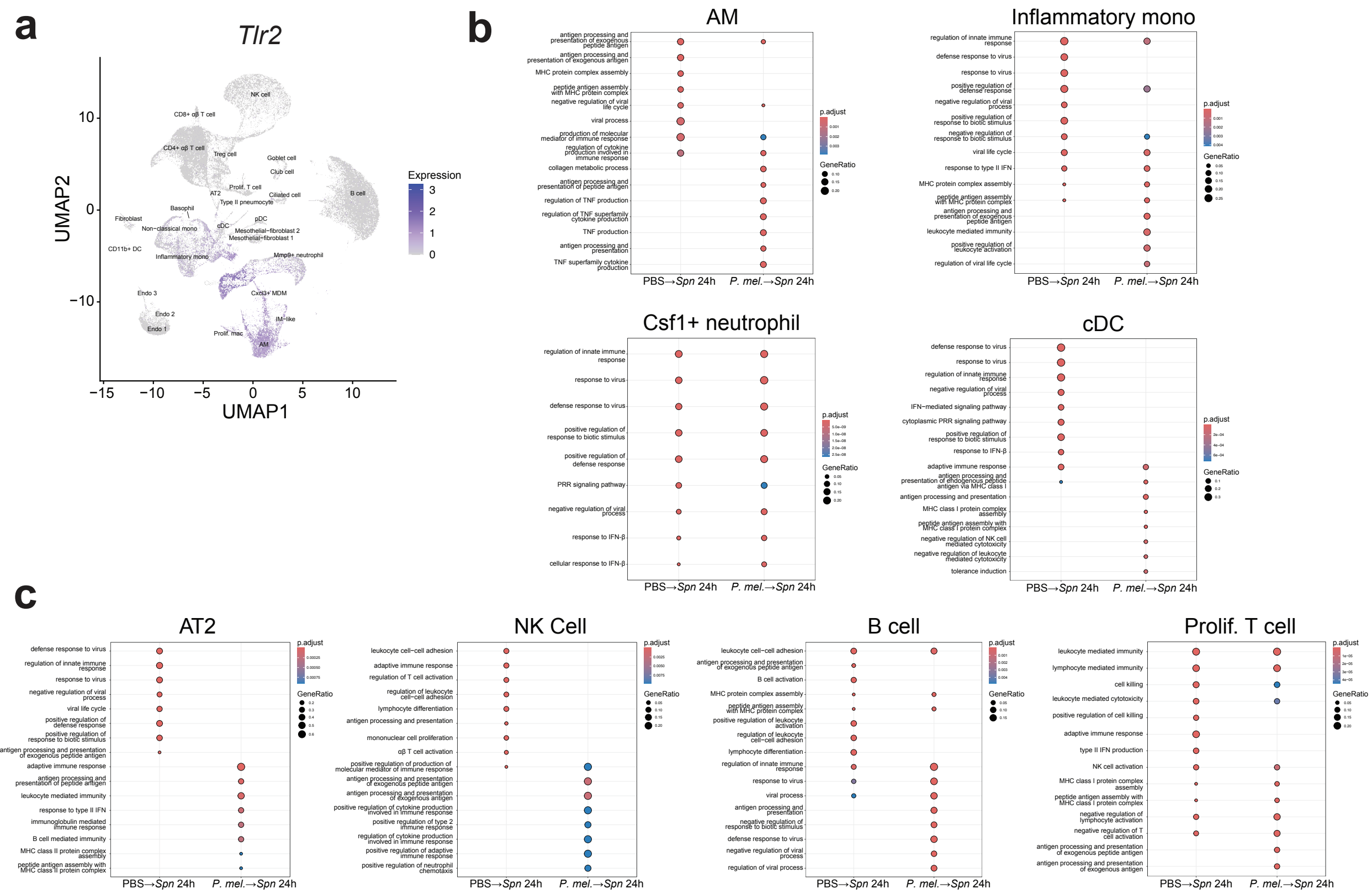

Figure S3: *P. melaninogenica* priming upregulates distinct immune pathways. (**A**) UMAP overlay of *Tlr2* gene expression. Unbiased pathway analysis indicating the top gene ontology (GO) terms for (**B**) selected myeloid cell populations and (**C**) select non-myeloid populations in *Spn*-infected mice with or without i.t. exposure to *P. mel* HK 24 hours prior to infection. Abbreviations: AM = alveolar macrophage, mono = monocyte, cDC = conventional DC, AT2 = alveolar type-2, NK = natural killer, Prolif. = proliferating.

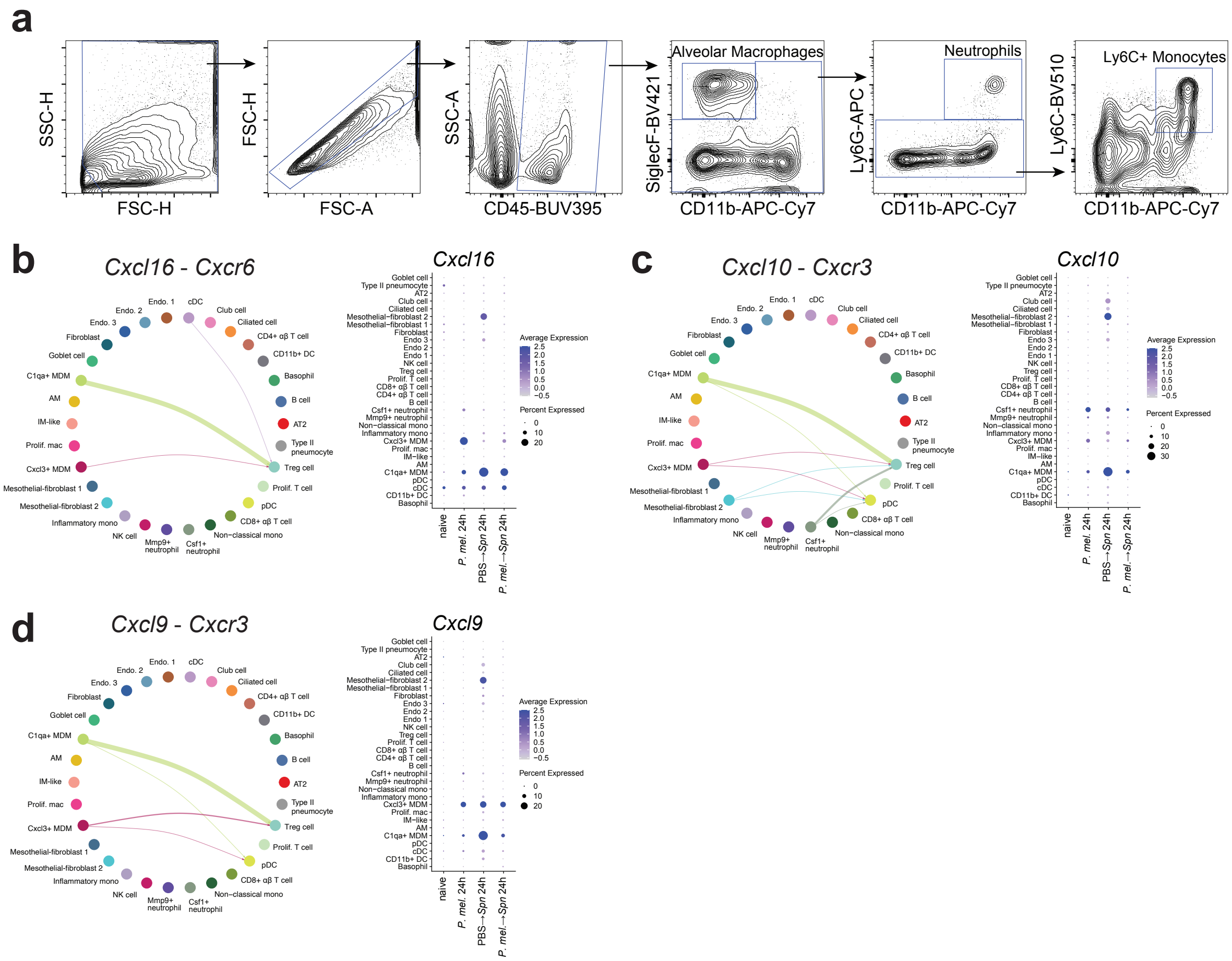
a
SSC-H
FSC-H
FSC-H
FSC-A
SSC-A
CD45-BUV395
SiglecF-BV421
CD11b-APC-Cy7
Alveolar Macrophages
Ly6G-APC
CD11b-APC-Cy7
Neutrophils
Ly6C-BV510
CD11b-APC-Cy7
Ly6C+ Monocytes
b
Cxcl16 - Cxcr6
Cxcl16
c
Cxcl10 - Cxcr3
Cxcl10
d
Cxcl9 - Cxcr3
Cxcl9
Average Expression
Percent Expressed
naive
P. mel. 24h
PBS→Spn 24h
P. mel.→Spn 24h
Endo. 1
cDC
Club cell
Ciliated cell
CD4+ αβ T cell
CD11b+ DC
Basophil
B cell
AT2
Type II pneumocyte
Treg cell
Prolif. T cell
pDC
CD8+ αβ T cell
Non-classical mono
Csf1+ neutrophil
Mmp9+ neutrophil
NK cell
Inflammatory mono
Mesothelial-fibroblast 2
Mesothelial-fibroblast 1
Cxcl3+ MDM
Prolif. mac
IM-like
AM
C1qa+ MDM
Goblet cell
Fibroblast
Endo. 3
Endo. 2

Figure S4: Macrophages signal to Tregs through chemokine expression.

(**A**) Flow cytometry gating strategy to identify AMs, neutrophils, and Ly6C+ monos/macs. Gating strategy was used for all flow cytometry experiments. CellChat circle plots of inferred cell-cell communication networks for *Cxcl16*-*Cxcr6* (**B**) *Cxcl10*-*Cxcr3* (**C**), and *Cxcr9*-*Cxcr3* (**D**) signaling along with dot plots showing expression of corresponding chemokine genes by condition. Abbreviations: AT2 = alveolar type-2, endo = endothelial, NK = natural killer, Treg = T regulatory cell, Prolif = proliferating, mono = monocyte, MDM = monocyte-derived macrophage, IM = interstitial macrophage, mac = macrophage, AM = alveolar macrophage, DC = dendritic cell, pDC = plasmacytoid DC, cDC = conventional DC.

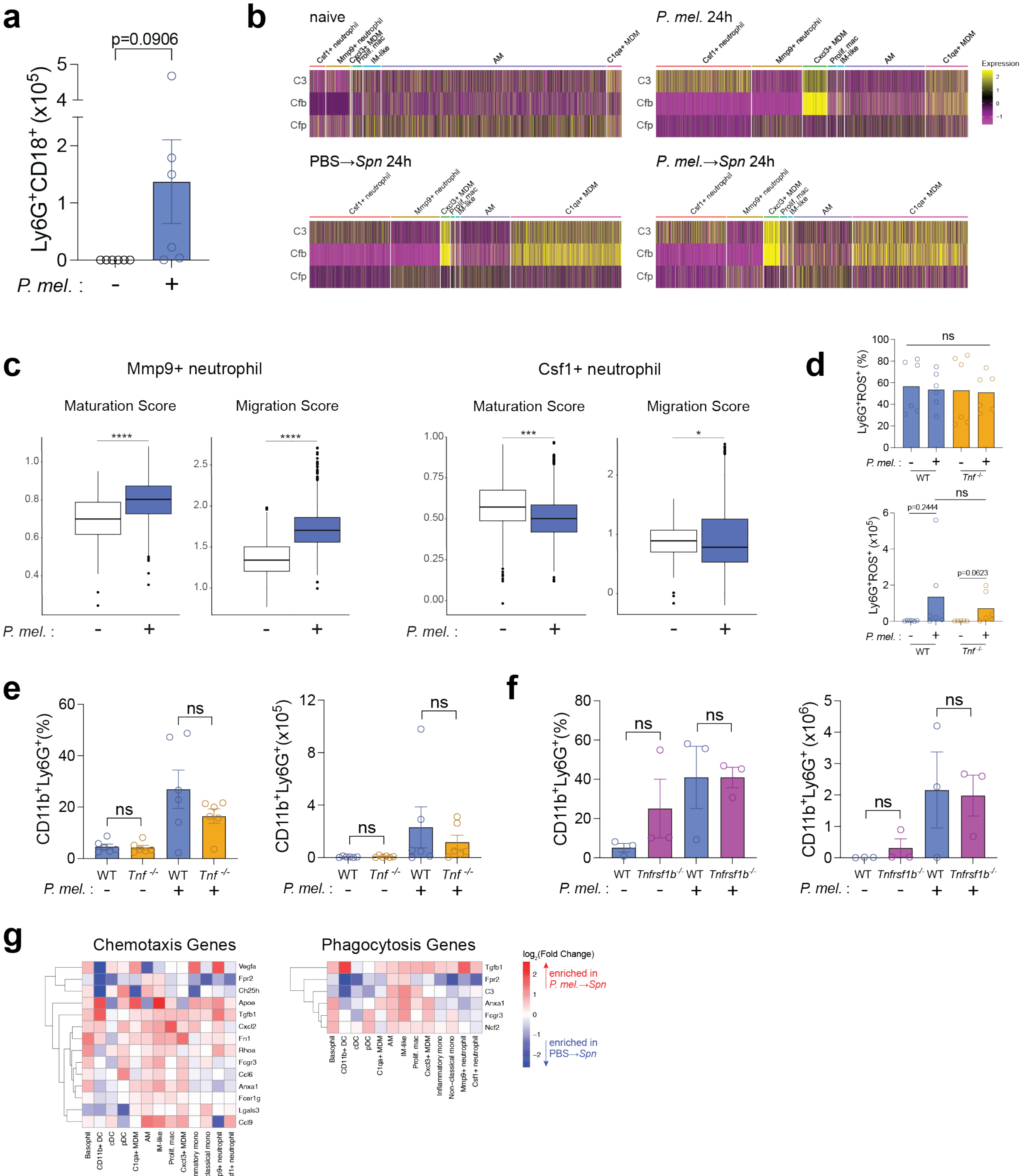

a
p=0.0906
Ly6G+CD18+ (x10^5)
P. mel. :
-
+
b
naive
P. mel. 24h
PBS→Spn 24h
P. mel.→Spn 24h
Csf1+ neutrophil
Mmp9+ neutrophil
Cxcl3+ MDM
Prolif. mac
IM-like
AM
C1qa+ MDM
C3
Cfb
Cfp
Expression
c
Mmp9+ neutrophil
Csf1+ neutrophil
Maturation Score
Migration Score
****
***
*
P. mel. :
d
Ly6G+ROS+ (%)
Ly6G+ROS+ (x10^5)
ns
p=0.2444
p=0.0623
WT
Tnf -/-
e
CD11b+Ly6G+(%)
CD11b+Ly6G+(x10^5)
ns
f
CD11b+Ly6G+(%)
CD11b+Ly6G+ (x10^6)
Tnfrsf1b-/-
g
Chemotaxis Genes
Phagocytosis Genes
Vegfa
Fpr2
Ch25h
Apoe
Tgfb1
Cxcl2
Fn1
Rhoa
Fcgr3
Ccl6
Anxa1
Fcer1g
Lgals3
Ccl9
C3
Ncf2
Basophil
CD11b+ DC
cDC
pDC
C1qa+ MDM
AM
IM-like
Prolif. mac
Cxcl3+ MDM
Inflammatory mono
Non-classical mono
Mmp9+ neutrophil
Csf1+ neutrophil
log2(Fold Change)
enriched in P. mel.→Spn
enriched in PBS→Spn

Figure S5: TNF signaling does not regulate neutrophil ROS production during *P. melaninogenica* exposure.

(**A**) Quantification of Ly6G+CD18+ neutrophil cell numbers in lung tissue of mice exposed to FITC-labeled *Spn* HK for 3 hours, with prior exposure to PBS or *P. mel* HK (mean ± SEM; n = 6 biological replicates pooled from 2 independent experiments). (**B**) Heatmap displaying alternative complement pathway gene expression across macrophage and neutrophil populations, split by condition. (**C**) Module scoring analysis of maturation and migration signature genes in neutrophil populations of naïve mice and mice exposed to *P. mel* HK. (**D**) Quantification of Ly6G+ROS+ neutrophils via flow cytometry in WT and *Tnf*$^{-/-}$ mice along with 24-hour exposure to PBS or *P. mel* HK (mean ± SEM; n = 6 biological replicates pooled from 2 independent experiments). Quantification of CD11b+Ly6G+ neutrophils via flow cytometry in WT vs *Tnf*$^{-/-}$ (**E**) or *Tnfrsf1b*$^{-/-}$ (**F**) mice exposed to PBS or *P. mel* HK (mean ± SEM; n = 3-6 biological replicates pooled from 1-2 experiments). (**G**) Heatmaps displaying log-fold changes of chemotaxis and phagocytosis gene expression in myeloid cells of mice exposed to *P. mel* HK prior to *Spn* infection, relative to mice infected with *Spn* alone. Abbreviations: AT2 = alveolar type-2, endo = endothelial, NK = natural killer, Treg = T regulatory cell, Prolif = proliferating, mono = monocyte, MDM = monocyte-derived macrophage, IM = interstitial macrophage, mac = macrophage, AM = alveolar macrophage, DC = dendritic cell, pDC = plasmacytoid DC, cDC = conventional DC. Data were analyzed by unpaired t test (**A**), Wilcoxon rank-sum test (**C**), or Kruskal-Wallis with Dunn's post hoc test (**D,E,F**). *p<0.05, ***p<0.001, ****p<0.0001.

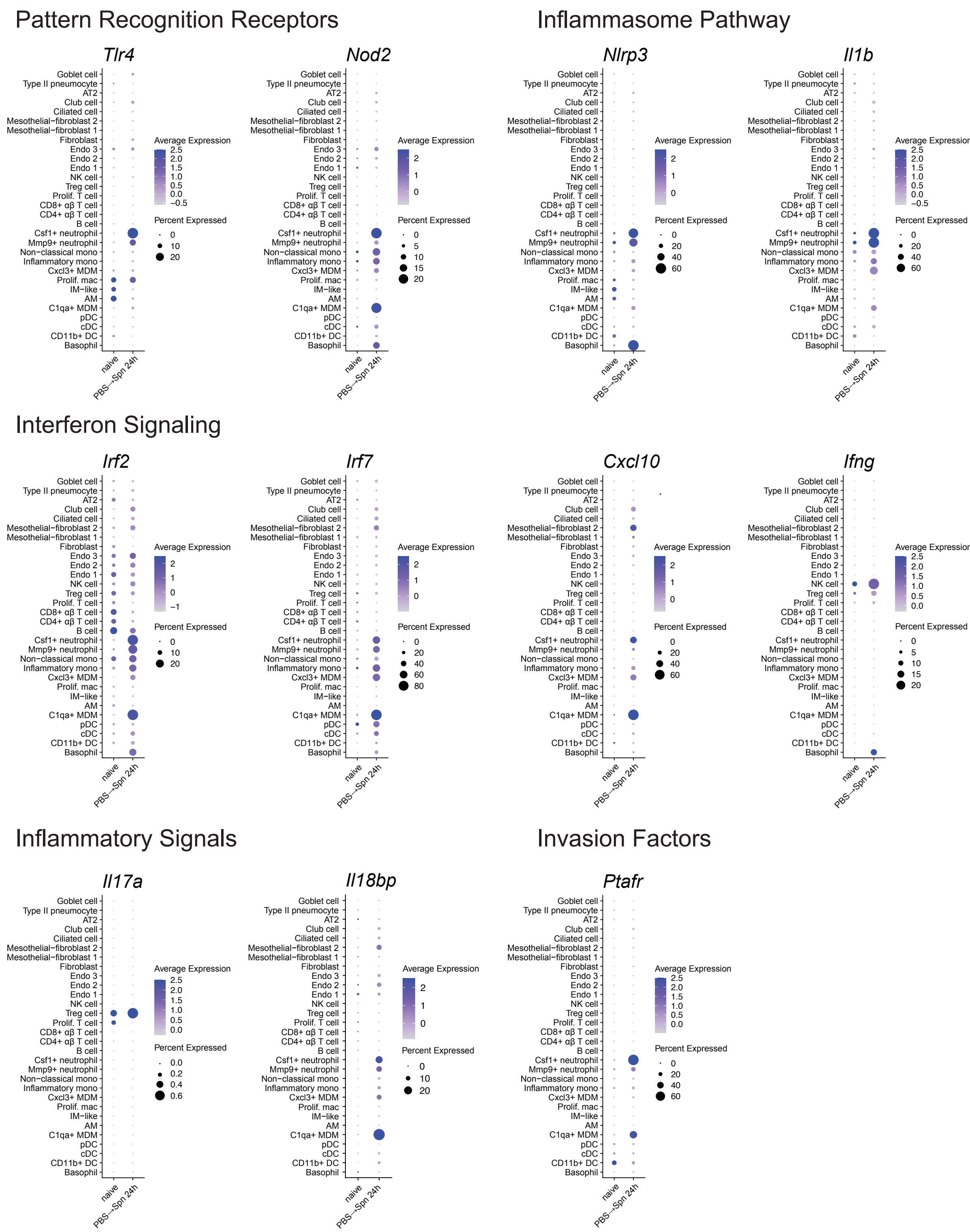

Pattern Recognition Receptors
Tlr4
Nod2
Inflammasome Pathway
Nlrp3
Il1b
Interferon Signaling
Irf2
Irf7
Cxcl10
Ifng
Inflammatory Signals
Il17a
Il18bp
Invasion Factors
Ptafr
Goblet cell
Type II pneumocyte
AT2
Club cell
Ciliated cell
Mesothelial−fibroblast 2
Mesothelial−fibroblast 1
Fibroblast
Endo 3
Endo 2
Endo 1
NK cell
Treg cell
Prolif. T cell
CD8+ αβ T cell
CD4+ αβ T cell
B cell
Csf1+ neutrophil
Mmp9+ neutrophil
Non−classical mono
Inflammatory mono
Cxcl3+ MDM
Prolif. mac
IM−like
AM
C1qa+ MDM
pDC
cDC
CD11b+ DC
Basophil
naive
PBS→Spn 24h
Average Expression
Percent Expressed

Figure S6: *S. pneumoniae* infection induces expression of canonical genes involved in host response to infection.

(**A**) Dot plots showing expression of genes involved in pattern recognition receptor signaling, inflammasome signaling, interferon signaling, inflammatory signaling, and host cell adhesion across all cell populations in naïve vs *Spn*-infected mice. (**B**) Dot plots showing expression of interferon receptor genes across all cell populations and all conditions. Abbreviations: AT2 = alveolar type-2, endo = endothelial, NK = natural killer, Treg = T regulatory cell, Prolif = proliferating, mono = monocyte, MDM = monocyte-derived macrophage, IM = interstitial macrophage, mac = macrophage, AM = alveolar macrophage, DC = dendritic cell, pDC = plasmacytoid DC, cDC = conventional DC.

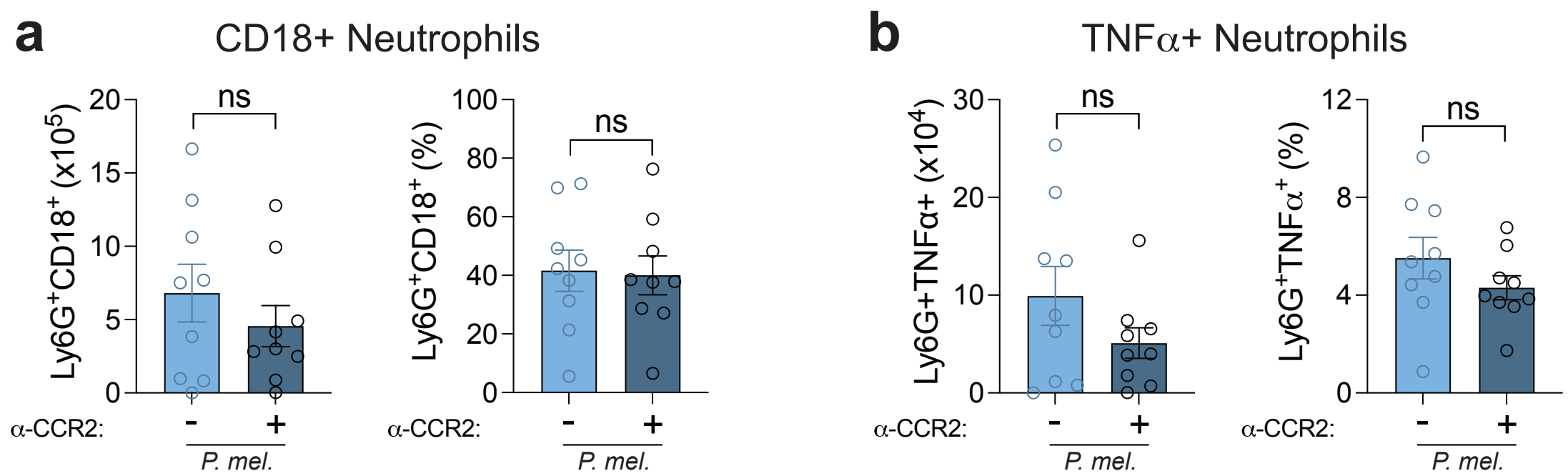

Figure S7: CCR2+ depletion does not impact neutrophil activation after *P. melaninogenica* exposure.

Flow cytometry quantification of (**A**) Ly6G+CD18+ neutrophils and (**B**) TNFα+ neutrophils in mice treated with isotype control or anti-CCR2 antibodies prior to *P. mel* HK exposure (mean ± SEM; n = 9 biological replicates pooled from 3 independent experiments). Data were analyzed by unpaired t test.
